# Host-similar fragments in the African swine fever virus genome: distribution, functions, and evolution

**DOI:** 10.1186/s13567-025-01539-3

**Published:** 2025-05-27

**Authors:** Zhaozhong Zhu, Na Li, Qin Sun, Xizi Long, Tao Wang, Hua-Ji Qiu

**Affiliations:** 1 School of Public Health, Hengyang Medical School, University of South China, Hengyang, 421001 China; 2 State Key Laboratory for Animal Disease Control and Prevention, National African Swine Fever Para-Reference Laboratory, National High Containment Facilities for Animal Diseases Control and Prevention, Harbin Veterinary Research Institute, Chinese Academy of Agricultural Sciences, Harbin, China

**Keywords:** African swine fever virus, host-similar fragments, similarity network, evolution, recombination

## Abstract

African swine fever virus (ASFV) predominantly infects *Argasidae* and *suids*, resulting in high morbidity and mortality in pigs. Despite the crucial role that viral sequences resembling those of the host play in the virus’s survival, there are limited comprehensive studies on the genomic similarities between ASFV and its hosts. Consequently, this study employs homology analysis to construct a similarity network between ASFV and its hosts (*Argasidae* and *suids*), investigating the distribution, function, evolution, and origins of these similar sequences in ASFV. Our findings indicate that the host-similar fragments are mainly distributed between positions 70000 and 180000 of the ASFV genome, primarily within non-coding regions. Notably, these non-coding fragments are often associated with promoter functions. Furthermore, the analysis of *suid* proteins that share similarities with ASFV proteins reveals that they predominantly exhibit RNA polymerase activity and are involved in metabolic processes. Evolutionary analysis indicates that pan-similar sequences of ASFV exist in an open state, highlighting the diversity of these analogous sequences. Additionally, a positive correlation was identified between the occurrence of recombination breakpoints and similar sequences, indicating that homologous recombination may serve as a crucial mechanism driving the formation of these analogous sequences.

**Supplementary Information:**

The online version contains supplementary material available at 10.1186/s13567-025-01539-3.

## Introduction

African swine fever virus (ASFV) is a large, double-stranded DNA virus that primarily infects *Argasidae* and *suids*, resulting in significant economic losses to the global pig industry [1–3]. The virus is maintained in a sylvatic cycle between ticks and warthogs in Africa, where it remains asymptomatic in warthogs but causes severe hemorrhagic fever in domestic pigs [4, 5]. Without an effective vaccine, control relies on quarantine, culling, and biosecurity [6, 7]. ASFV has a large genome (170**–**194 kb) that encodes over 150 proteins, most of which are poorly understood [8, 9]. Furthermore, ASFV can be classified into approximately 24 genotypes and is primarily found in sub-Saharan Africa [10]. Its ability to evade the immune system and high mutation rate complicates vaccine development [11, 12].

All viral sequences that resemble those of the host, indicating genetic similarities between viruses and their hosts, play crucial roles in viral infection and pathogenesis [13–15]. For example, *Flaviviruses*, such as dengue and Zika viruses, exploit specific sequences similar to those of the host to manipulate the host’s immune response, resulting in viral proliferation and pathogenesis [13]. Human papillomavirus (HPV), a DNA virus, uses its similarities to human DNA to infect and replicate [16]. Additionally, some HPV proteins, like E6 and E7, mimic human tumour suppressors, disrupting cell growth control and increasing the risk of cancer [16]. Over 100 sequence similarities exist between SARS-CoV-2 and the human genome, suggesting that the virus may have undergone extensive genetic exchange during its evolution [17]. Some host similarity sequences facilitate genetic exchange through horizontal gene transfer, influencing the evolution of both bacterial and bacteriophage genomes [15, 18]. Studying all virus-host-similar sequences is crucial for understanding viral pathogenesis, developing antiviral strategies, and elucidating the broader implications of viral infections on host biology [15, 17].

Several studies have examined the sequence similarities between ASFV and its hosts [19, 20], with one notably finding ASFV-like integrated (ASFLI) elements in the genome of *Ornithodoros moubata*. This discovery suggests a co-evolutionary relationship that dates back over 1.47 million years [19]. These elements, which comprise 10% of the ASFV genome, may contribute to RNA interference-based protection in *O. moubata* [19]. Additionally, ASFV shows a preference for A/U-ending codons, contrasting with its hosts’ G/C preference, indicating an adaptation strategy to evade immune responses [20]. This research enhances our understanding of ASFV’s evolution and host interactions, which are crucial for developing control strategies [19, 20].

Studying virus-host similarity sequences helps unravel the complex interactions between viruses and their hosts [21, 22]. For example, phages acquire host sequences to enhance infection efficiency and alter their host range [21, 22]. However, research on the similarities between ASFV and host sequences is still limited. This study addresses this gap by identifying and analysing these sequences’ distribution, functions, and evolution in ASFV. The findings deepen our understanding of ASFV genetic diversity and evolution, offering new strategies for antiviral measures and vaccine development.

## Materials and methods

### Identification of the host-similar ASFV fragments

A total of 446 ASFV genome sequences were downloaded from the NCBI Genome database [23]. To obtain high-quality ASFV genomes, we removed all incomplete and missing genome sequences. The redundant genome sequences were eliminated at a 95% similarity threshold using CD-HIT [24]. We retained 73 complete and non-redundant ASFV genomes (Additional file 1). The *Argasidae* and *suid* genomes were downloaded from the NCBI nucleotide database [25]. To identify fragments in the ASFV genome that were similar to the host, including *Argasidae* and *suids*, BLAST (version 2.9.0) [26] was used to search the ASFV genomes. ASFV genome fragments with nucleotide sequence identities greater than 95% were considered host-similar.

### Annotations of the ASFV genomes

The genes and proteins of ASFV were predicted in 73 ASFV genomes using the GeneMarkS server [27] with its default parameters. The predicted ASFV proteins were blasted against all ASFV proteins downloaded from the NCBI RefSeq database to infer protein function [28]. Predicted proteins with a sequence identity exceeding 30%, coverage greater than 40%, and e-values less than 0.001 were identified as ASFV proteins. The functional categorisation of the ASFV proteins was adapted to those outlined in Alí et al. [29].

### Identification of the suid proteins similar to ASFV proteins

All *suid* proteins were obtained from the RefSeq database. A BLAST analysis was conducted to identify the *suid* proteins homologous to ASFV by comparing the annotated ASFV proteins from 73 genomes with the *suid* proteins. The *suid* proteins with a sequence identity ≥ 30% and e-value ≤ 0.001 were considered potentially similar to ASFV proteins.

### Functional analysis of the host-similar ASFV fragments

The promoter function of ASFV fragments was predicted using BDGP [30].

### Functional analysis of the suid proteins

The Kyoto Encyclopedia of Genes and Genomes (KEGG) pathway and gene ontology (GO) enrichment analysis were performed using the enrichKEGG and enrichGO functions from the clusterProfiler package (version 4.6.2) [31] in R (version 4.2.2) [32]. The pathways and GO terms with *q*-values below 0.05 were considered significantly enriched.

### Phylogenetic trees and ASFV genotyping

To determine ASFV genotypes, a maximum likelihood phylogenetic tree was constructed using the C-terminal sequence (415 bp) of the p72 gene from 73 ASFV isolates [10]. The tree was generated using MEGA (version X) [33] with default parameters. To assess the reliability of the phylogenetic tree, we performed bootstrap analysis with 100 replicates. Genotypes were assigned based on established criteria from previous studies, and the resulting phylogeny was visualised using Dendroscope (version 3.8.8) [34].

### Pan-genomic analysis of the host-similar ASFV fragments

All host-similar ASFV fragments were grouped into clusters based on 100% sequence identity. PanGP [35], employing DG sampling algorithms, was used to visualise the characteristic curves of both pan-host-similar ASFV fragments and core-host-similar ASFV fragments. In R software, the ‘plotrix’ package (version 4.2.2) [36] generated a flower plot illustrating the distribution of core, dispensable, and unique host-similar ASFV fragment clusters.

### Identification of the recombination events in the ASFV genome

To detect recombination events in the aligned ASFV genomes, we employed RDP (version 5) [37], including GENECONV, Bootscan, Maxchi, Chimaera, SiSscan, PhylPro, and 3Seq. These methods were applied with default parameters, and only recombination events with a significant *p*-value < 0.05 were considered. For each detected event, RDP provided information on the recombination region, recombination breakpoints, the recombinant virus, the potential major and minor parent viruses, and the support from each method. Only events detected by at least two methods were used for subsequent analysis to ensure robustness (Additional file 2).

### Statistical analysis

All statistical analyses were conducted in R (version 4.2.2) [32]. The Wilcoxon rank-sum test was performed using the wilcox.test function; linear regression was fitted with the lm function, and the correlation coefficient was calculated using the cor.test function.

## Results

### Identification of similar fragments between the ASFV and host genomes

**Figure 1:**
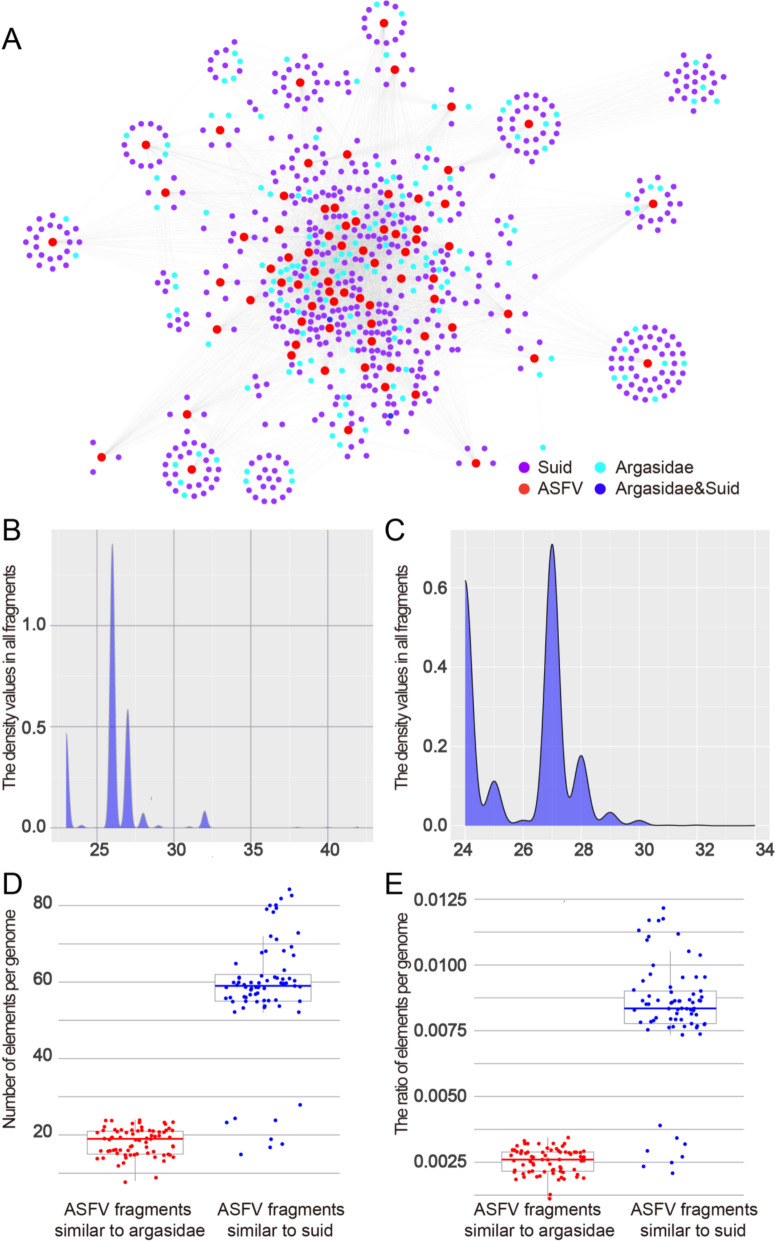
O**verview of the similarity network between ASFV and its hosts (Argasidae and suids). A** ASFV similarity network with *Argasidae* and *suids.* **B** The length of the ASFV fragments similar to *Argasidae.* **C** The length of the ASFV fragments similar to *suids.* **D** Number of the ASFV fragments similar to *Argasidae* and *suids* per genome. **E** The ratio of the ASFV fragments similar to *Argasidae* and *suids* per genome.

Seventy-three high-quality ASFV genome sequences were obtained from the NCBI Genome database. The ASFV genomes were found to contain a large number of fragments similar to those of the hosts (host-similar fragments), including *Argasidae* and *suids*. A similarity network was constructed using a 95% sequence identity threshold (Figure 1A). In total, 147 *Argasidae*-similar fragments and 578 *suid*-similar fragments were identified. Notably, two ASFV fragments exhibited similarity to sequences in both *suid* and *Argasidae* genomes.

The analysis examined the length, number, and distribution ratio of the host-similar fragments in the ASFV genome. The lengths of *Argasidae*-similar fragments ranged from 23 to 42 (median: 26; Figure 1B). In contrast, the lengths of *suid*-similar fragments ranged from 24 to 34 (median: 27; Figure 1C). The number of *Argasidae*-similar fragments per genome varied from 8 to 24 (median: 19; Figure 1D). *Suid*-similar fragments ranged from 15 to 84 (median: 59; Figure 1D). The ratio of *Argasidae*-similar fragments ranged from 0.0011 to 0.0034 (median: 0.0026; Figure 1E), whereas *suid*-similar fragments ranged from 0.0021 to 0.0122 (median: 0.0084; Figure 1E). *Argasidae*-similar fragments were shorter, fewer, and had lower ratios, suggesting that ASFV may have acquired more *suid*-similar sequences during its adaptation from *Argasidae* to *suids*.

### Distribution of the host-similar fragments in the ASFV genome

**Figure 2:**
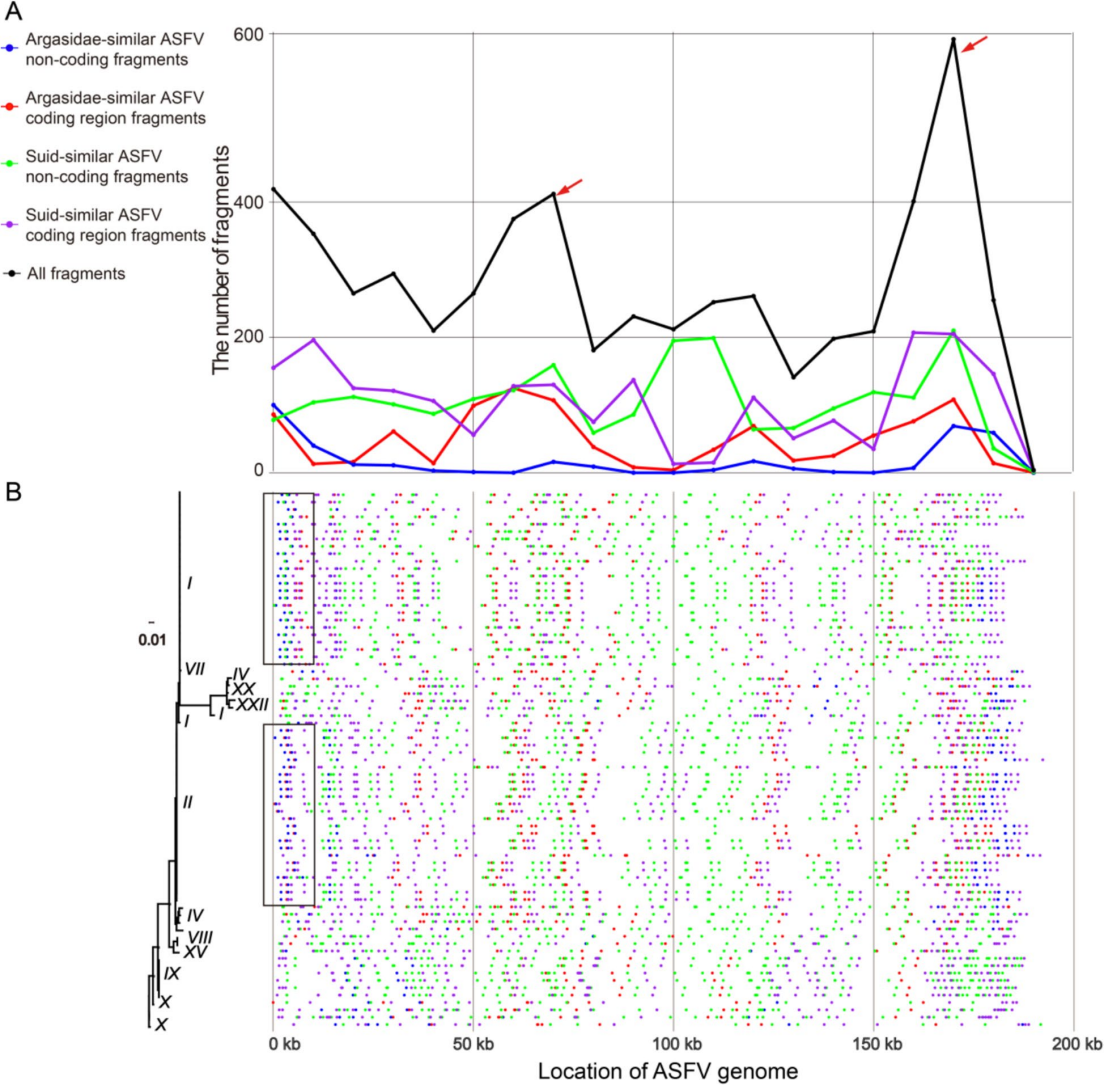
**The number and positions of the host-similar fragments in the ASFV genome**. **A** The number of host-similar ASFV fragments per kb in the 73 ASFV genomes. **B** Phylogenetic tree (left) based on the C-terminal sequence of p72 (415 bp) and the genotype labels (Greek numerals). The positions of the host-similar ASFV fragments (right) with genotype-specific examples are highlighted (black frames).

We analysed the distribution within the viral genome to determine the location of host-similar ASFV fragments and subsequently calculated their occurrences per 10 kb window of ASFV genomes. Specifically, the median of *Argasidae*-similar ASFV coding region fragments, *Argasidae*-similar ASFV non-coding fragments, *suid*-similar ASFV coding region fragments, and *suid*-similar ASFV non-coding region fragments per 10 kb window of the ASFV genome were 36, 6.5, 102.5, and 116, respectively. Interestingly, two prominent hotspots were identified at window positions 70 kb and 180 kb (Figure 2A, red arrows). Furthermore, among the ten ASFV genotypes we identified, some host-similar ASFV fragments were identified as genotype-specific (Figure 2B, black box). This finding enriches our understanding of ASFV genetic diversity and reveals specific adaptive mechanisms involved in its evolutionary process.

### Functional and sequence characteristics of the host-similar ASFV fragments

**Figure 3:**
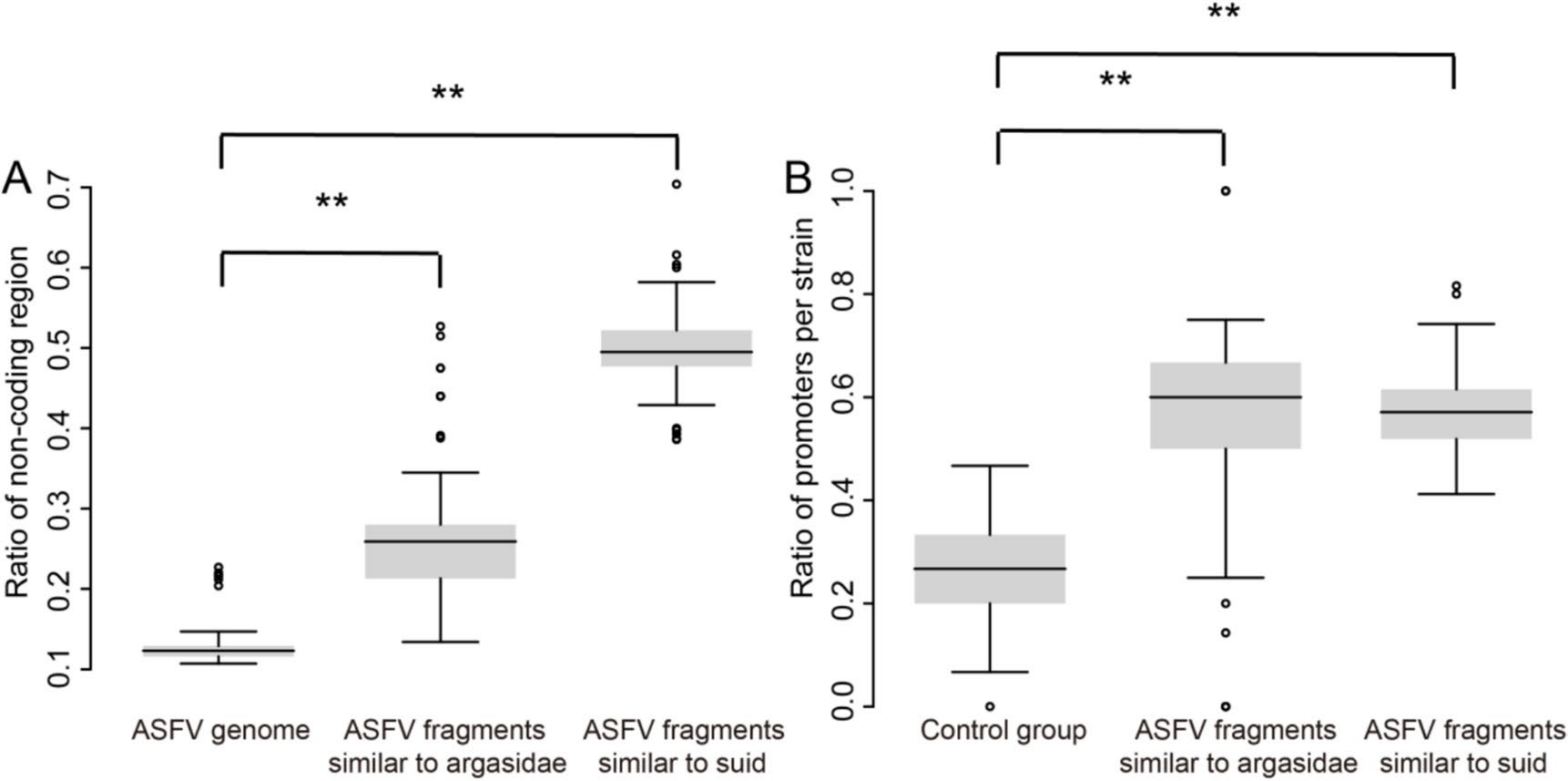
**The functions of the host-similar fragments in the non-coding regions in the ASFV genome. A** Distribution of the non-coding regions in the ASFV genome, *Argasidae*-similar, and *suid*-similar fragments. **B** The ratio of the promoters in the host-similar (*Argasidae* and *suids*) vs the non-host-similar fragments of the ASFV genome. ‘**’, *p* < 0.01.

These fragments were found more frequently in non-coding regions than in ASFV gene sequences (Figure 3A). Approximately 50% of *Argasidae*-similar fragments and 26% of *suid*-similar fragments were located in non-coding regions. In contrast, only 12% of the entire ASFV genome was found in non-coding regions. Given the abundance of host-similar ASFV fragments in non-coding regions, we explored their potential role in transcriptional regulation. Previous research indicates that viral fragments resembling host genes frequently contain promoter sequences, aiding immune evasion and adaptation to the host [38].

We calculated the ratio of promoters within the host-similar sequences in the ASFV non-coding regions using randomly selected non-coding sequences as a control (see Materials and methods). The results showed significantly higher promoter ratios in *Argasidae*- and *suid*-similar non-coding fragments compared with the control group (Figure 3B). This finding suggests that these fragments may contribute to ASFV transcriptional regulation by enriching promoter elements, thereby influencing its life cycle and pathogenicity. Additionally, we analysed the GC content of host-similar sequences in coding and non-coding regions (Additional file 3). These fragments exhibited significantly lower GC content than the overall genome, suggesting they may have undergone higher mutation rates during viral evolution.

### Functions of the suid proteins similar to the ASFV proteins

**Figure 4:**
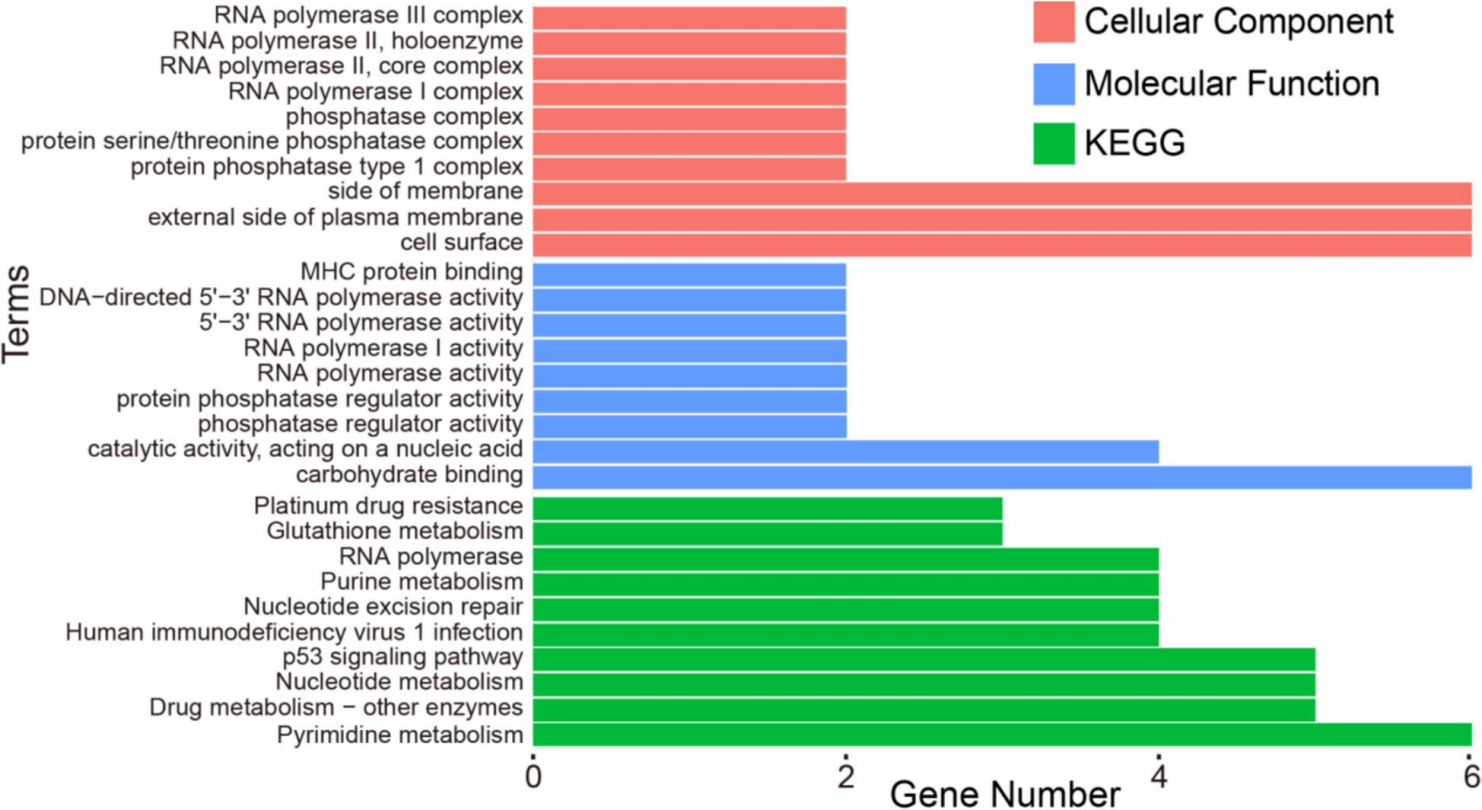
**Functional analysis of the suid proteins similar to the ASFV proteins.** The top 10 enriched terms in the *suid* proteins within the domains of biological processes, cellular components, molecular functions, and KEGG pathways.

We conducted GO and KEGG pathway enrichment analyses on *suid* proteins similar to ASFV proteins to investigate the biological processes in *suid* cells that ASFV may disrupt by mimicking these proteins (see Materials and methods). We identified 42 *suid* proteins that share sequence similarity with ASFV proteins. Functional enrichment analysis revealed that most enriched GO terms were closely associated with the RNA polymerase complex in the cellular component category (Figure 4). Moreover, most enriched GO terms were related to RNA polymerase activity in the molecular function category, including 5'−3'RNA polymerase, RNA polymerase I activity, and DNA-directed 5'−3'RNA polymerase activity. Four of the ten enriched KEGG pathways were also linked to metabolic processes, such as glutathione and purine metabolism. These results emphasise the significance of RNA polymerase complexes and metabolic pathways in the functions of these *suid* proteins.

We also performed a functional analysis of ASFV proteins similar to *suid* proteins, revealing that most ASFV proteins had unknown functions, followed by those involved in structure and morphogenesis (Additional file 4). These findings suggest that ASFV may disrupt host cell processes by mimicking proteins involved in host structure and morphogenesis, thereby supporting viral survival.

### Pan-genomic analysis of the host-similar ASFV fragments

**Figure 5:**
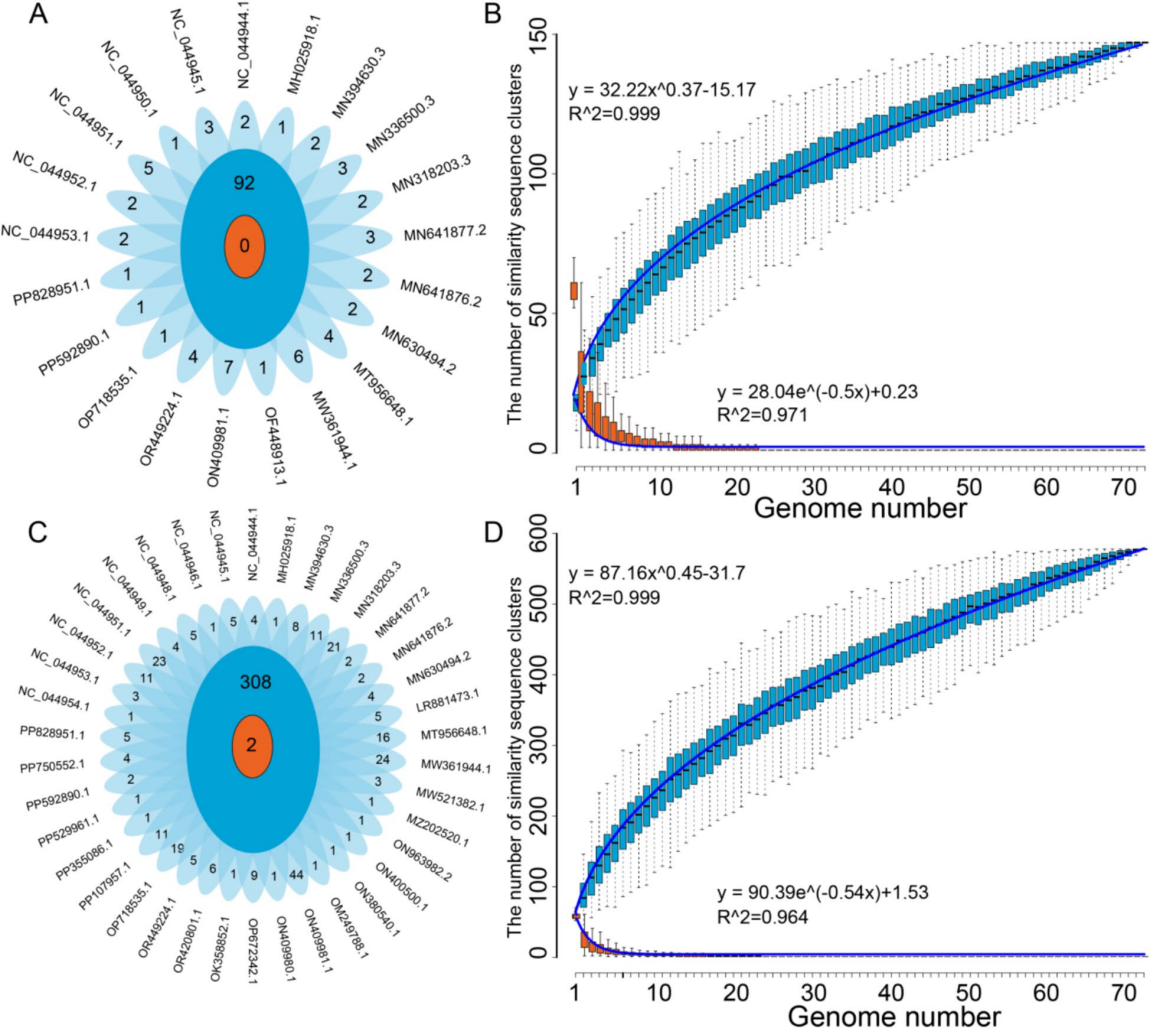
**Pan-genome analysis of the host-similar fragments in the ASFV genome. A** A flower plot depicting the core, dispensable, and strain-specific ASFV fragments similar to *Argasidae* across the 73 ASFV strains. The diagram depicts the core (in the centre), dispensable (in the annulus), and strain-specific (in the petals) fragment number for the 73 ASFV strains. **B** The profile of the pan-ASFV and core fragments similar to *Argasidae*. The exponent (0.37) of the pan-genome curve is greater than zero, indicating an open pan-genome. **C** A flower plot depicting the core, dispensable, and strain-specific ASFV fragments similar to *suids* across 73 ASFV strains. The diagram depicts the core fragments number (in the centre), the dispensable fragments number (in the annulus), and the strain-specific fragments number (in the petals) for the 73 ASFV strains. **D** The profile of the pan-ASFV fragments and core fragments similar to *suids*. The exponent (0.45) of the pan-genome curve is greater than zero, indicating an open pan-genome.

A pan-genomic analysis of the host-similar ASFV fragments was carried out, during which all host-similar ASFV fragments in all ASFV genomes were collectively defined as ‘pan-ASFV fragments’. These were categorised into two types: core ASFV fragment clusters, which appear in every genome, and non-core ASFV fragments, which were found in one or more but not all genomes. The non-core fragments appearing only in one genome are considered unique ASFV fragments. Analysis of the pan-ASFV fragments similar to *Argasidae* revealed no core fragment clusters but identified 147 non-core fragment clusters, including 55 unique fragments (Figure 5A). Similarly, analysis of the *suid*-similar pan-fragments identified two core fragment clusters and 576 non-core fragment clusters, including 268 unique fragments (Figure 5C).

A power-law regression model was applied to fit the number of dispensable ASFV fragment clusters analogous to those found in *Argasidae* (Figure 5B) and *suids* (Figure 5D). The models, y = 32.22x^0.37^–15.17 and y = 87.16x^0.45^–31.7, accurately captured this relationship, respectively. As more ASFV genomes were analysed, dispensable host-similar fragment clusters steadily increased. This trend indicates ASFV’s adaptability, likely due to the expansion of pan-fragments with each additional genome, which can generate new host-similar fragment clusters.

An exponential model was used to fit the number of core ASFV fragment clusters similar to *Argasidae* (Figure 5B) and *suids* (Figure 5D). The models, y = 28.04e^(−0.5x)^ + 0.23 and y = 90.39e^(−0.54x)^ + 1.53, accurately described this trend. As the number of the genomes analysed increased, the core ASFV fragment clusters significantly declined. This outcome denotes the diverse and dynamic nature of the host-similar fragment within the ASFV genome.

### The relationship between the host-similar ASFV fragments and the ASFV homologous recombination

**Figure 6:**
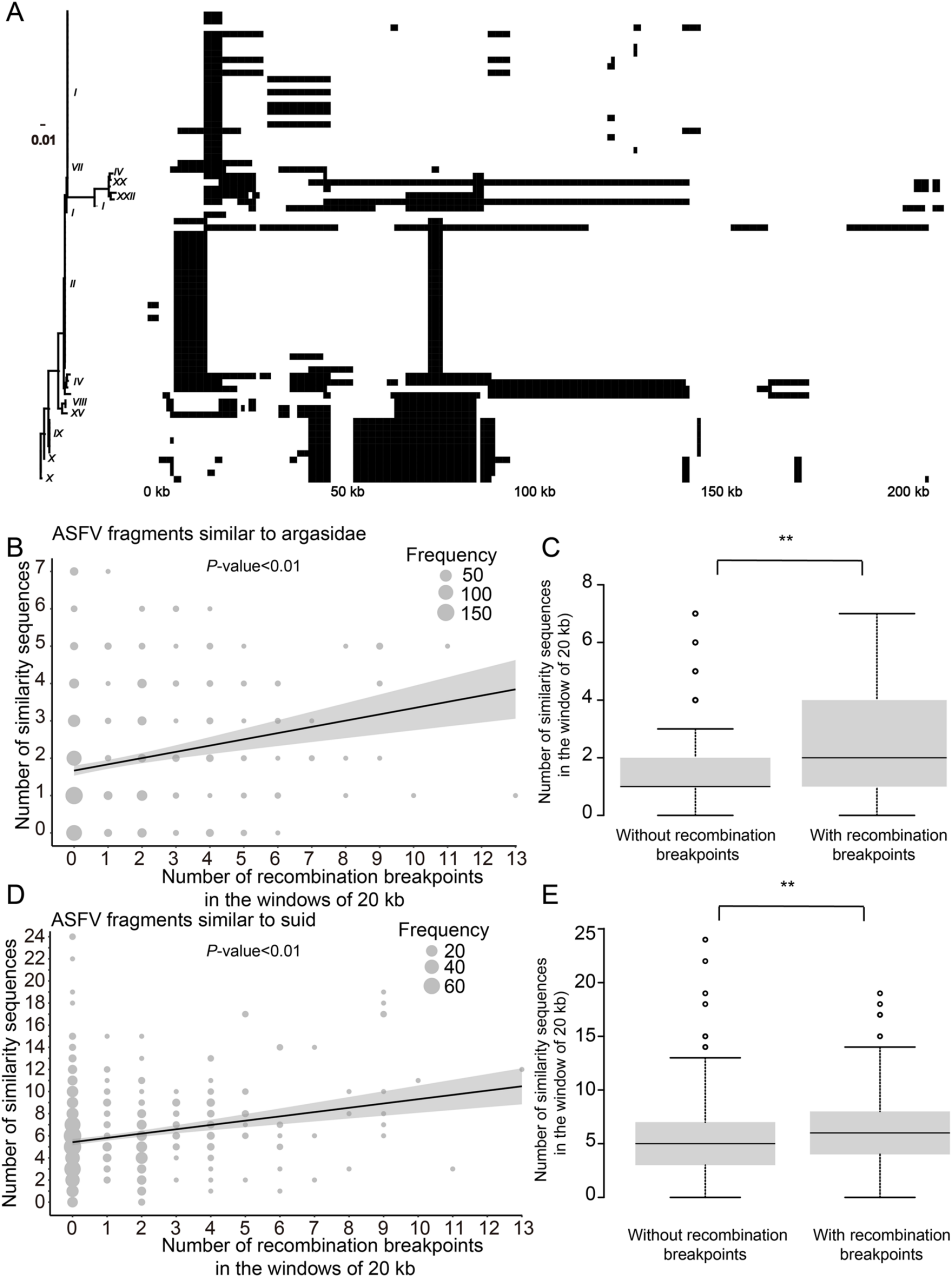
**The relationship between the recombination breakpoints and the host-similar fragments in the ASFV genome. A** The recombination regions (in black) in the ASFV genome. **B** The number of the recombination breakpoints in a sliding window of 20 kb *versus* the number of *Argasidae*-similar ASFV fragments. **C** Comparison of the number of the *Argasidae*-similar ASFV fragments in a sliding window of 20 kb with and without the recombination breakpoints in the ASFV genome. **D** The number of recombination breakpoints in a sliding window of 20 kb versus the number of *suid*-similar ASFV fragments. **E** Comparison of the number of *suid*-similar ASFV fragments in a sliding window of 20 kb with and without the recombination breakpoints in the ASFV genome. ‘**’, *p* < 0.01.

Studies suggest that homologous recombination may facilitate the exchange of nucleic acid sequences between viruses and their hosts. Therefore, we examined the effect of homologous recombination on host similarity fragments in ASFV. Recombination events were observed across the 73 ASFV genomes, with each genome undergoing between 1 and 17 recombination events (median: 3; Figure 6A). Interestingly, the number of recombination breakpoints identified in each 20 kb ASFV genome window was positively correlated with the number of *Argasidae*-similar ASFV fragments (Figure 6B) and *suid*-similar ASFV fragments (Figure 6D) identified in the same region, with Pearson correlation coefficients of 0.19 and 0.21, respectively. Specifically, the median number of *Argasidae*-similar fragments in windows containing recombination breakpoints was 1, while *suid*-similar fragments had a median of 5. In contrast, windows without recombination breakpoints had medians of 2 for the *Argasidae*-similar fragments (Figure 6C) and 6 for the *suid*-similar fragments (Figure 6E).

## Discussion

The research highlights a significant discovery of fragments in ASFV genomes that closely resemble those of their hosts, *Argasidae and suids*. This similarity suggests a complex evolutionary history involving potential genetic exchange or recombination. These host-similar fragments were primarily distributed in non-coding regions and were more inclined to include promoter regions. Additionally, *suid* proteins share similarities with ASFV proteins, mainly functioning as RNA polymerases and participating in metabolic processes. Such findings may prompt further investigation into the virus’s adaptability and ability to influence the host’s immune response, providing valuable insights into ASFV’s pathogenicity and host range.

Our research has identified numerous similar sequences between the ASFV and its host, consistent with previous studies [19]. These sequences, likely resulting from nucleic acid exchanges between the virus and host, provide the virus with advantages in infection and survival, enhancing its adaptability and pathogenicity. Moreover, some ASFV protein fragments are similar to RNA polymerase and metabolism-related proteins in pigs. Therefore, these fragments may interfere with essential functions such as transcription and metabolism, providing a viral advantage. They may also help viruses evade the host’s immune system by mimicking host proteins and facilitating replication and transmission within host cells. This process underscores the complexity of host-virus interactions and the necessity for a thorough understanding to develop effective countermeasures against ASFV.

The pan-genomic analysis of host-similar ASFV fragments provides valuable insights into the virus’s genetic diversity and adaptability. Identifying core and non-core fragment clusters, including unique ones, emphasises the genome’s dynamic nature. The power-law regression model shows a steady increase in dispensable fragment clusters with each genome analysed, reflecting the virus’s capability to expand its genetic repertoire and adapt to new hosts [39, 40]. In contrast, the exponential model suggests a decline in essential core fragment clusters as more genomes are studied, underscoring the virus’s flexibility and potential for rapid evolution.

Exploring the relationship between host-similar ASFV fragments and homologous recombination events provides a compelling narrative of the virus’s genetic plasticity. The positive correlation between recombination breakpoints and the presence of host-similar sequences suggests a mechanism through which the virus may acquire and integrate host genetic material. This finding is particularly significant as it implies that recombination may contribute to the virus’s ability to adapt and evolve, potentially leading to new strains with altered virulence or host range. The study’s insights into the role of recombination in shaping the ASFV genome contribute to a more nuanced understanding of viral evolution and the complexities of host-virus co-evolution [41].

However, it is important to note that this study has two limitations. First, the size and number of similar sequences in the ASFV genome may be influenced by the chosen similarity threshold. We applied a 95% similarity threshold to ensure the authenticity of the sequences, as using a lower threshold could identify more sequences but might also increase false positives. Second, the predicted promoter functions within the ASFV-similar fragments need further validation. In future studies, experimental approaches, such as reporter assays or mutagenesis analyses, could be employed to validate the predicted promoter activities.

## Supplementary Information

**Additional file 1:** **The ASFV strains used in this study.**

**Additional file 2:** **The recombination events detected by at least two methods in RDP5. **The results detected by RDP5 listed the recombination regions, recombinant viruses, potential major and minor parents, and the methods supporting the events.

**Additional file 3:** **The GC content of host-similar fragments in the coding and non-coding regions of the ASFV genome.**

**Additional file 4:** **The functions of the ASFV proteins similar to those of the Argasidaeand suidproteins.**

## Data Availability

Datasets used and/or analyzed during the current study are available from the corresponding author on reasonable request.
