# Supplementary material for: Host-similar fragments in the African swine fever virus genome: distribution, functions, and evolution
Source: Vet Res. 2025 May 27;56:108. doi: 10.1186/s13567-025-01539-3 (PMC12107907; doi:10.1186/s13567-025-01539-3)
Supplement: Supplementary file 1 — Additional file 1: The ASFV strains used in this study. [file 13567_2025_1539_MOESM1_ESM.docx]

| Accession number | Size(bp) | GC% | Host | Database |
| --- | --- | --- | --- | --- |
| NC_044941.1 | 182362 | 38.61 | Swine,Argasidae | NCBI Assembly/Genome |
| NC_044942.1 | 180365 | 38.81 | Swine,Argasidae | NCBI Assembly/Genome |
| NC_044943.1 | 172051 | 38.87 | Swine,Argasidae | NCBI Assembly/Genome |
| NC_044944.1 | 193886 | 38.37 | Swine,Argasidae | NCBI Assembly/Genome |
| NC_044945.1 | 191058 | 38.25 | Swine,Argasidae | NCBI Assembly/Genome |
| NC_044946.1 | 184368 | 38.53 | Swine,Argasidae | NCBI Assembly/Genome |
| NC_044948.1 | 189333 | 38.42 | Swine,Argasidae | NCBI Assembly/Genome |
| NC_044949.1 | 186528 | 38.58 | Swine,Argasidae | NCBI Assembly/Genome |
| NC_044950.1 | 190773 | 38.45 | Swine,Argasidae | NCBI Assembly/Genome |
| NC_044951.1 | 185689 | 38.62 | Swine,Argasidae | NCBI Assembly/Genome |
| NC_044952.1 | 190324 | 38.43 | Swine,Argasidae | NCBI Assembly/Genome |
| NC_044953.1 | 192714 | 38.37 | Swine,Argasidae | NCBI Assembly/Genome |
| NC_044954.1 | 187612 | 37.97 | Swine,Argasidae | NCBI Assembly/Genome |
| NC_044955.1 | 184638 | 38.48 | Swine,Argasidae | NCBI Assembly/Genome |
| NC_044956.1 | 182284 | 38.59 | Swine,Argasidae | NCBI Assembly/Genome |
| NC_001659.2 | 170101 | 38.95 | Swine,Argasidae | NCBI Assembly/Genome |
| PP810980.1 | 185379 | 38.59 | Swine,Argasidae | NCBI Assembly/Genome |
| PP828951.1 | 187717 | 38.45 | Swine,Argasidae | NCBI Assembly/Genome |
| OR660089.1 | 189844 | 38.38 | Swine,Argasidae | NCBI Assembly/Genome |
| OR371517.1 | 184953 | 38.48 | Swine,Argasidae | NCBI Assembly/Genome |
| PP348677.1 | 182459 | 38.69 | Swine,Argasidae | NCBI Assembly/Genome |
| PP750552.1 | 185346 | 38.56 | Swine,Argasidae | NCBI Assembly/Genome |
| OQ504954.1 | 185395 | 38.59 | Swine,Argasidae | NCBI Assembly/Genome |
| OQ504955.1 | 185342 | 38.6 | Swine,Argasidae | NCBI Assembly/Genome |
| OQ504956.1 | 185431 | 38.59 | Swine,Argasidae | NCBI Assembly/Genome |
| PP592890.1 | 182530 | 38.56 | Swine,Argasidae | NCBI Assembly/Genome |
| PP529961.1 | 184187 | 38.6 | Swine,Argasidae | NCBI Assembly/Genome |
| PP478517.1 | 185620 | 38.58 | Swine,Argasidae | NCBI Assembly/Genome |
| PP355086.1 | 181087 | 38.53 | Swine,Argasidae | NCBI Assembly/Genome |
| OZ005801.1 | 187015 | 38.5 | Swine,Argasidae | NCBI Assembly/Genome |
| PP107957.1 | 185514 | 37.93 | Swine,Argasidae | NCBI Assembly/Genome |
| OZ003747.1 | 185088 | 38.52 | Swine,Argasidae | NCBI Assembly/Genome |
| OP479889.1 | 184733 | 38.55 | Swine,Argasidae | NCBI Assembly/Genome |
| OP718535.1 | 184651 | 38.55 | Swine,Argasidae | NCBI Assembly/Genome |
| OR449224.1 | 213885 | 38.01 | Swine,Argasidae | NCBI Assembly/Genome |
| OR420801.1 | 188854 | 38.53 | Swine,Argasidae | NCBI Assembly/Genome |
| OR387520.1 | 183179 | 38.56 | Swine,Argasidae | NCBI Assembly/Genome |
| OR290104.2 | 189416 | 38.4 | Swine,Argasidae | NCBI Assembly/Genome |
| OR227304.1 | 189607 | 38.38 | Swine,Argasidae | NCBI Assembly/Genome |
| OR180113.1 | 192224 | 38.33 | Swine,Argasidae | NCBI Assembly/Genome |
| OK358852.1 | 192298 | 38.31 | Swine,Argasidae | NCBI Assembly/Genome |
| OP672342.1 | 185316 | 38.53 | Swine,Argasidae | NCBI Assembly/Genome |
| ON409979.1 | 184521 | 38.5 | Swine,Argasidae | NCBI Assembly/Genome |
| ON409980.1 | 182514 | 38.61 | Swine,Argasidae | NCBI Assembly/Genome |
| ON409981.1 | 178830 | 38.15 | Swine,Argasidae | NCBI Assembly/Genome |
| ON409983.1 | 183389 | 38.54 | Swine,Argasidae | NCBI Assembly/Genome |
| OF448913.1 | 185493 | 38.4 | Swine,Argasidae | NCBI Assembly/Genome |
| OM249788.1 | 180891 | 38.51 | Swine,Argasidae | NCBI Assembly/Genome |
| OM105586.1 | 188389 | 38.5 | Swine,Argasidae | NCBI Assembly/Genome |
| ON380539.1 | 189737 | 38.38 | Swine,Argasidae | NCBI Assembly/Genome |
| ON380540.1 | 189456 | 38.39 | Swine,Argasidae | NCBI Assembly/Genome |
| MZ566623.1 | 189148 | 38.7 | Swine,Argasidae | NCBI Assembly/Genome |
| ON400500.1 | 178014 | 38.79 | Swine,Argasidae | NCBI Assembly/Genome |
| ON963982.2 | 192265 | 38.33 | Swine,Argasidae | NCBI Assembly/Genome |
| OL310288.1 | 188072 | 38.42 | Swine,Argasidae | NCBI Assembly/Genome |
| MZ945536.1 | 171235 | 38.84 | Swine,Argasidae | NCBI Assembly/Genome |
| MZ945537.1 | 172025 | 38.85 | Swine,Argasidae | NCBI Assembly/Genome |
| MW736602.1 | 181813 | 38.57 | Swine,Argasidae | NCBI Assembly/Genome |
| MZ202520.1 | 189523 | 38.43 | Swine,Argasidae | NCBI Assembly/Genome |
| MW521382.1 | 188643 | 38.48 | Swine,Argasidae | NCBI Assembly/Genome |
| MW656282.1 | 189355 | 38.4 | Swine,Argasidae | NCBI Assembly/Genome |
| MW361944.1 | 186471 | 38.47 | Swine,Argasidae | NCBI Assembly/Genome |
| MT956648.1 | 180916 | 38.53 | Swine,Argasidae | NCBI Assembly/Genome |
| LR881473.1 | 192206 | 38.43 | Swine,Argasidae | NCBI Assembly/Genome |
| MN270972.1 | 183723 | 38.55 | Swine,Argasidae | NCBI Assembly/Genome |
| MN270973.1 | 181816 | 38.58 | Swine,Argasidae | NCBI Assembly/Genome |
| MN630494.2 | 184820 | 39.24 | Swine,Argasidae | NCBI Assembly/Genome |
| MN641876.2 | 187621 | 39.2 | Swine,Argasidae | NCBI Assembly/Genome |
| MN641877.2 | 189903 | 39.06 | Swine,Argasidae | NCBI Assembly/Genome |
| MN318203.3 | 183292 | 39.16 | Swine,Argasidae | NCBI Assembly/Genome |
| MN336500.3 | 190066 | 39.12 | Swine,Argasidae | NCBI Assembly/Genome |
| MN394630.3 | 188458 | 39.19 | Swine,Argasidae | NCBI Assembly/Genome |
| MH025918.1 | 188630 | 38.47 | Swine,Argasidae | NCBI Assembly/Genome |
