# Supplementary material for: Host-similar fragments in the African swine fever virus genome: distribution, functions, and evolution
Source: Vet Res. 2025 May 27;56:108. doi: 10.1186/s13567-025-01539-3 (PMC12107907; doi:10.1186/s13567-025-01539-3)
Supplement: Supplementary file 2 — Additional file 2: The recombination events detected by at least two methods in RDP5. The results detected by RDP5 listed the recombination regions, recombinant viruses, potential major and minor parents, and the methods supporting the events. [file 13567_2025_1539_MOESM2_ESM.docx]

|  | **Breakpoint Positions** |  |  |  |  |  |
| --- | --- | --- | --- | --- | --- | --- |
| **In Alignment** |  | **In Recombinant Sequence** |  |  |  |  |
| **Begin** | **End** | **Begin** | **End** | **Recombinant Sequence(s)** | **Minor Parental Sequence(s)** | **Major Parental Sequence(s)** |
| 9481 | 25607 | 6885 | 8830 | NC_001659.2 | Unknown (MN394630.3) | MN270972.1 |
|  |  |  |  | NC_044942.1[P] | Unknown(MN641876.2) | NC_044941.1 |
|  |  |  |  |  | Unknown(MN318203.3) | NC_044953.1 |
|  |  |  |  |  | Unknown(MN336500.3) | NC_044955.1 |
|  |  |  |  |  |  | NC_044956.1 |
|  |  |  |  |  |  | OR371517.1 |
|  |  |  |  |  |  | PP592890.1 |
|  |  |  |  |  |  | OR387520.1 |
|  |  |  |  |  |  | OM249788.1 |
|  |  |  |  |  |  | MW736602.1 |
|  |  |  |  |  |  | MZ202520.1 |
|  |  |  |  |  |  | MN270973.1 |
|  |  |  |  |  |  |  |
| 33715 | 49873 | 19403 | 33443 | ^PP810980.1 | OR449224.1 | Unknown (NC_044942.1) |
|  |  |  |  |  |  | Unknown(NC_044941.1) |
|  |  |  |  |  |  | Unknown(NC_044955.1) |
|  |  |  |  |  |  | Unknown(NC_044956.1) |
|  |  |  |  |  |  | Unknown(NC_001659.2) |
|  |  |  |  |  |  | Unknown(OR371517.1) |
|  |  |  |  |  |  | Unknown(PP592890.1) |
|  |  |  |  |  |  | Unknown(OR387520.1) |
|  |  |  |  |  |  | Unknown(MW736602.1) |
|  |  |  |  |  |  | Unknown(MN270972.1) |
|  |  |  |  |  |  | Unknown(MN270973.1) |
|  |  |  |  |  |  |  |
| 33715 | 49873 | 19289 | 33329 | ^OQ504954.1 | ON400500.1 | Unknown (NC_044941.1) |
|  |  |  |  |  |  | Unknown(NC_044942.1) |
|  |  |  |  |  |  | Unknown(NC_044943.1) |
|  |  |  |  |  |  | Unknown(NC_044955.1) |
|  |  |  |  |  |  | Unknown(NC_044956.1) |
|  |  |  |  |  |  | Unknown(NC_001659.2) |
|  |  |  |  |  |  | Unknown(OR371517.1) |
|  |  |  |  |  |  | Unknown(PP592890.1) |
|  |  |  |  |  |  | Unknown(OR387520.1) |
|  |  |  |  |  |  | Unknown(MZ945536.1) |
|  |  |  |  |  |  | Unknown(MZ945537.1) |
|  |  |  |  |  |  | Unknown(MW736602.1) |
|  |  |  |  |  |  | Unknown(MN270972.1) |
|  |  |  |  |  |  | Unknown(MN270973.1) |
|  |  |  |  |  |  |  |
| 33715 | 49873 | 19295 | 33335 | ^OQ504956.1 | ON400500.1 | Unknown (NC_044941.1) |
|  |  |  |  |  |  | Unknown(NC_044942.1) |
|  |  |  |  |  |  | Unknown(NC_044943.1) |
|  |  |  |  |  |  | Unknown(NC_044955.1) |
|  |  |  |  |  |  | Unknown(NC_044956.1) |
|  |  |  |  |  |  | Unknown(NC_001659.2) |
|  |  |  |  |  |  | Unknown(OR371517.1) |
|  |  |  |  |  |  | Unknown(PP592890.1) |
|  |  |  |  |  |  | Unknown(OR387520.1) |
|  |  |  |  |  |  | Unknown(MZ945537.1) |
|  |  |  |  |  |  | Unknown(MW736602.1) |
|  |  |  |  |  |  | Unknown(MN270972.1) |
|  |  |  |  |  |  | Unknown(MN270973.1) |
|  |  |  |  |  |  |  |
| 33715 | 49873 | 19394 | 33434 | PP478517.1 | ON400500.1 | Unknown (NC_044941.1) |
|  |  |  |  |  |  | Unknown(NC_044942.1) |
|  |  |  |  |  |  | Unknown(NC_044943.1) |
|  |  |  |  |  |  | Unknown(NC_044955.1) |
|  |  |  |  |  |  | Unknown(NC_044956.1) |
|  |  |  |  |  |  | Unknown(NC_001659.2) |
|  |  |  |  |  |  | Unknown(OR371517.1) |
|  |  |  |  |  |  | Unknown(PP592890.1) |
|  |  |  |  |  |  | Unknown(OR387520.1) |
|  |  |  |  |  |  | Unknown(MZ945536.1) |
|  |  |  |  |  |  | Unknown(MZ945537.1) |
|  |  |  |  |  |  | Unknown(MW736602.1) |
|  |  |  |  |  |  | Unknown(MN270972.1) |
|  |  |  |  |  |  | Unknown(MN270973.1) |
|  |  |  |  |  |  |  |
| 231411* | 7902 | 185969* | 4631 | ^MW361944.1 | PP810980.1 | Unknown (ON409980.1) |
|  |  |  |  |  |  | Unknown(NC_044944.1) |
|  |  |  |  |  |  | Unknown(NC_044946.1) |
|  |  |  |  |  |  | Unknown(NC_044954.1) |
|  |  |  |  |  |  | Unknown(PP750552.1) |
|  |  |  |  |  |  | Unknown(OZ005801.1) |
|  |  |  |  |  |  | Unknown(PP107957.1) |
|  |  |  |  |  |  | Unknown(OZ003747.1) |
|  |  |  |  |  |  | Unknown(MZ566623.1) |
|  |  |  |  |  |  | Unknown(MT956648.1) |
|  |  |  |  |  |  | Unknown(MH025918.1) |
|  |  |  |  |  |  |  |
| 67698 | 118753 | 46746 | 94734 | ^MW361944.1 | MZ202520.1 | Unknown (ON409981.1) |
|  |  |  |  |  | NC_044941.1 |  |
|  |  |  |  |  | NC_044942.1 |  |
|  |  |  |  |  | NC_044943.1 |  |
|  |  |  |  |  | NC_044949.1 |  |
|  |  |  |  |  | NC_044950.1 |  |
|  |  |  |  |  | NC_044951.1 |  |
|  |  |  |  |  | NC_044952.1 |  |
|  |  |  |  |  | NC_044953.1 |  |
|  |  |  |  |  | NC_044955.1 |  |
|  |  |  |  |  | NC_044956.1 |  |
|  |  |  |  |  | NC_001659.2 |  |
|  |  |  |  |  | PP828951.1 |  |
|  |  |  |  |  | OR371517.1 |  |
|  |  |  |  |  | PP592890.1 |  |
|  |  |  |  |  | OR420801.1 |  |
|  |  |  |  |  | OR387520.1 |  |
|  |  |  |  |  | OM249788.1 |  |
|  |  |  |  |  | MZ945536.1 |  |
|  |  |  |  |  | MZ945537.1 |  |
|  |  |  |  |  | MW736602.1 |  |
|  |  |  |  |  | LR881473.1 |  |
|  |  |  |  |  | MN270972.1 |  |
|  |  |  |  |  | MN270973.1 |  |
|  |  |  |  |  | MN630494.2 |  |
|  |  |  |  |  | MN641876.2 |  |
|  |  |  |  |  | MN641877.2 |  |
|  |  |  |  |  | MN318203.3 |  |
|  |  |  |  |  | MN394630.3 |  |
|  |  |  |  |  |  |  |
| 39327 | 47314 | 27951 | 30304 | ^PP529961.1 | Unknown (PP348677.1) | NC_044954.1 |
|  |  |  |  |  |  | NC_044944.1 |
|  |  |  |  |  |  | NC_044945.1 |
|  |  |  |  |  |  | NC_044946.1 |
|  |  |  |  |  |  | PP750552.1 |
|  |  |  |  |  |  | OZ005801.1 |
|  |  |  |  |  |  | PP107957.1 |
|  |  |  |  |  |  | OZ003747.1 |
|  |  |  |  |  |  | ON409980.1 |
|  |  |  |  |  |  | ON409981.1 |
|  |  |  |  |  |  | MZ566623.1 |
|  |  |  |  |  |  | MT956648.1 |
|  |  |  |  |  |  | MH025918.1 |
|  |  |  |  |  |  |  |
| 31979 | 51504 | 20072 | 36814 | ^MW361944.1 | ON400500.1 | Unknown (NC_044946.1) |
|  |  |  |  |  | PP529961.1 | Unknown(NC_044944.1) |
|  |  |  |  |  | PP355086.1 | Unknown(NC_044945.1) |
|  |  |  |  |  | OR449224.1 | Unknown(PP750552.1) |
|  |  |  |  |  | ON409983.1 | Unknown(OZ005801.1) |
|  |  |  |  |  | OF448913.1 | Unknown(OZ003747.1) |
|  |  |  |  |  | OM105586.1 | Unknown(ON409980.1) |
|  |  |  |  |  | ON380540.1 | Unknown(MZ566623.1) |
|  |  |  |  |  | OL310288.1 | Unknown(MT956648.1) |
|  |  |  |  |  |  | Unknown(MH025918.1) |
|  |  |  |  |  |  |  |
| 188685 | 209556 | 153174 | 172158 | ^MW361944.1 | OP672342.1 | Unknown (NC_044946.1) |
|  |  |  |  |  | NC_044948.1 | Unknown(NC_044944.1) |
|  |  |  |  |  | OR660089.1 | Unknown(NC_044945.1) |
|  |  |  |  |  | PP529961.1 | Unknown(PP750552.1) |
|  |  |  |  |  | PP355086.1 | Unknown(OZ005801.1) |
|  |  |  |  |  | OP479889.1 | Unknown(OZ003747.1) |
|  |  |  |  |  | OP718535.1 | Unknown(ON409980.1) |
|  |  |  |  |  | OR449224.1 | Unknown(MZ566623.1) |
|  |  |  |  |  | OR290104.2 | Unknown(MT956648.1) |
|  |  |  |  |  | OR227304.1 | Unknown(MH025918.1) |
|  |  |  |  |  | OR180113.1 |  |
|  |  |  |  |  | OK358852.1 |  |
|  |  |  |  |  | ON409979.1 |  |
|  |  |  |  |  | ON409983.1 |  |
|  |  |  |  |  | OF448913.1 |  |
|  |  |  |  |  | OM105586.1 |  |
|  |  |  |  |  | ON380539.1 |  |
|  |  |  |  |  | ON380540.1 |  |
|  |  |  |  |  | ON400500.1 |  |
|  |  |  |  |  | ON963982.2 |  |
|  |  |  |  |  | OL310288.1 |  |
|  |  |  |  |  | MW521382.1 |  |
|  |  |  |  |  | MW656282.1 |  |
|  |  |  |  |  |  |  |
| 16582 | 29226 | 10896 | 17018 | ^MN641876.2 | Unknown (MW361944.1) | NC_044943.1 |
|  |  |  |  | NC_044949.1 |  | PP529961.1 |
|  |  |  |  | MN630494.2 |  | PP355086.1 |
|  |  |  |  |  |  | ON409983.1 |
|  |  |  |  |  |  | OF448913.1 |
|  |  |  |  |  |  | OM105586.1 |
|  |  |  |  |  |  | ON380540.1 |
|  |  |  |  |  |  | OL310288.1 |
|  |  |  |  |  |  |  |
| 16588 | 29214 | 10877 | 18842 | MW361944.1 | PP529961.1 | Unknown (MN641877.2) |
|  |  |  |  |  | PP355086.1 | Unknown(NC_044952.1) |
|  |  |  |  |  | ON409983.1 |  |
|  |  |  |  |  | OF448913.1 |  |
|  |  |  |  |  | OM105586.1 |  |
|  |  |  |  |  | ON380540.1 |  |
|  |  |  |  |  | OL310288.1 |  |
|  |  |  |  |  |  |  |
| 225912 | 230926* | 183525 | 185877* | ^MW361944.1 | ON963982.2 | Unknown (OM249788.1) |
|  |  |  |  |  | NC_044948.1 | Unknown(NC_044942.1) |
|  |  |  |  |  | PP529961.1 | Unknown(NC_044955.1) |
|  |  |  |  |  | PP355086.1 | Unknown(NC_001659.2) |
|  |  |  |  |  | OP479889.1 | Unknown(OR371517.1) |
|  |  |  |  |  | OP718535.1 | Unknown(OR387520.1) |
|  |  |  |  |  | OR290104.2 | Unknown(MZ202520.1) |
|  |  |  |  |  | OR180113.1 | Unknown(MN270972.1) |
|  |  |  |  |  | OK358852.1 |  |
|  |  |  |  |  | OF448913.1 |  |
|  |  |  |  |  | OM105586.1 |  |
|  |  |  |  |  | ON380539.1 |  |
|  |  |  |  |  | ON380540.1 |  |
|  |  |  |  |  | OL310288.1 |  |
|  |  |  |  |  | MW656282.1 |  |
|  |  |  |  |  |  |  |
| 157339 | 166185 | 125800 | 134551 | MW361944.1 | PP355086.1 | Unknown (MN394630.3) |
|  |  |  |  |  | NC_044948.1 | Unknown(MN630494.2) |
|  |  |  |  |  | OR660089.1 | Unknown(MN641876.2) |
|  |  |  |  |  | OR449224.1 | Unknown(MN641877.2) |
|  |  |  |  |  | OR290104.2 | Unknown(MN318203.3) |
|  |  |  |  |  | OR227304.1 | Unknown(MN336500.3) |
|  |  |  |  |  | OR180113.1 |  |
|  |  |  |  |  | OK358852.1 |  |
|  |  |  |  |  | OF448913.1 |  |
|  |  |  |  |  | OM105586.1 |  |
|  |  |  |  |  | ON380540.1 |  |
|  |  |  |  |  | ON963982.2 |  |
|  |  |  |  |  | OL310288.1 |  |
|  |  |  |  |  | MW656282.1 |  |
|  |  |  |  |  |  |  |
| 92796 | 94952 | 71537 | 73167 | ^MN394630.3 | ON409979.1 | NC_044950.1 |
|  |  |  |  |  | NC_044948.1 |  |
|  |  |  |  |  | OR660089.1 |  |
|  |  |  |  |  | PP529961.1 |  |
|  |  |  |  |  | PP355086.1 |  |
|  |  |  |  |  | OR449224.1 |  |
|  |  |  |  |  | OR290104.2 |  |
|  |  |  |  |  | OR227304.1 |  |
|  |  |  |  |  | OR180113.1 |  |
|  |  |  |  |  | OK358852.1 |  |
|  |  |  |  |  | OF448913.1 |  |
|  |  |  |  |  | ON380539.1 |  |
|  |  |  |  |  | ON380540.1 |  |
|  |  |  |  |  | ON963982.2 |  |
|  |  |  |  |  | OL310288.1 |  |
|  |  |  |  |  | MW656282.1 |  |
|  |  |  |  |  |  |  |
| 225334 | 230867 | 187597 | 190533 | ^NC_044953.1 | MN336500.3 | MZ202520.1 |
|  |  |  |  |  |  | NC_001659.2 |
|  |  |  |  |  |  | OR371517.1 |
|  |  |  |  |  |  | OM249788.1 |
|  |  |  |  |  |  |  |
| 33715 | 49873 | 16510 | 30550 | ^PP348677.1 | ON400500.1 | Unknown (NC_044955.1) |
|  |  |  |  |  |  | Unknown(NC_044941.1) |
|  |  |  |  |  |  | Unknown(NC_044942.1) |
|  |  |  |  |  |  | Unknown(NC_044943.1) |
|  |  |  |  |  |  | Unknown(NC_044956.1) |
|  |  |  |  |  |  | Unknown(NC_001659.2) |
|  |  |  |  |  |  | Unknown(OR371517.1) |
|  |  |  |  |  |  | Unknown(PP592890.1) |
|  |  |  |  |  |  | Unknown(OR387520.1) |
|  |  |  |  |  |  | Unknown(MZ945536.1) |
|  |  |  |  |  |  | Unknown(MZ945537.1) |
|  |  |  |  |  |  | Unknown(MW736602.1) |
|  |  |  |  |  |  | Unknown(MN270972.1) |
|  |  |  |  |  |  | Unknown(MN270973.1) |
|  |  |  |  |  |  |  |
| 144284 | 9480* | 117918 | 7389* | ^PP828951.1 | NC_044950.1 | NC_001659.2 |
|  |  |  |  |  |  | NC_044941.1 |
|  |  |  |  |  |  | NC_044955.1 |
|  |  |  |  |  |  | NC_044956.1 |
|  |  |  |  |  |  | OR371517.1 |
|  |  |  |  |  |  | PP592890.1 |
|  |  |  |  |  |  | OR387520.1 |
|  |  |  |  |  |  | MW736602.1 |
|  |  |  |  |  |  | MN270972.1 |
|  |  |  |  |  |  | MN270973.1 |
|  |  |  |  |  |  |  |
| 90057 | 91096* | 66884 | 67920* | ^NC_044946.1 | Unknown (NC_044953.1) | MN318203.3 |
|  |  |  |  | NC_044944.1[P] |  | MN641876.2 |
|  |  |  |  | NC_044945.1[P] |  | MN336500.3 |
|  |  |  |  | PP750552.1[P] |  |  |
|  |  |  |  | OZ005801.1 |  |  |
|  |  |  |  | OZ003747.1 |  |  |
|  |  |  |  | ON409980.1 |  |  |
|  |  |  |  | MZ566623.1 |  |  |
|  |  |  |  | MT956648.1[P] |  |  |
|  |  |  |  | MH025918.1 |  |  |
|  |  |  |  |  |  |  |
| 88330 | 90056* | 65623 | 67349* | MN318203.3 | Unknown (NC_044941.1) | NC_044944.1 |
|  |  |  |  | MN630494.2[P] | Unknown(NC_044943.1) | NC_044945.1 |
|  |  |  |  | MN641876.2[P] | Unknown(NC_044955.1) | NC_044946.1 |
|  |  |  |  | MN641877.2[P] | Unknown(NC_044956.1) | PP750552.1 |
|  |  |  |  | MN336500.3[P] | Unknown(OR371517.1) | ON409980.1 |
|  |  |  |  | MN394630.3[P] | Unknown(PP592890.1) | ON409981.1 |
|  |  |  |  |  | Unknown(OR387520.1) | MH025918.1 |
|  |  |  |  |  | Unknown(MW736602.1) |  |
|  |  |  |  |  | Unknown(MN270972.1) |  |
|  |  |  |  |  | Unknown(MN270973.1) |  |
|  |  |  |  |  |  |  |
| 67578 | 88329* | 49663 | 69279* | ^NC_044945.1 | Unknown (MN270972.1) | MN318203.3 |
|  |  |  |  | NC_044944.1 |  | MN336500.3 |
|  |  |  |  | NC_044946.1 |  |  |
|  |  |  |  | NC_044954.1[P] |  |  |
|  |  |  |  | PP750552.1 |  |  |
|  |  |  |  | OZ005801.1[P] |  |  |
|  |  |  |  | PP107957.1[P] |  |  |
|  |  |  |  | OZ003747.1[P] |  |  |
|  |  |  |  | ON409980.1 |  |  |
|  |  |  |  | ON409981.1 |  |  |
|  |  |  |  | MZ566623.1[P] |  |  |
|  |  |  |  | MT956648.1 |  |  |
|  |  |  |  | MH025918.1 |  |  |
|  |  |  |  |  |  |  |
| 92660 | 92765* | 73304 | 73409* | ^NC_044945.1 | Unknown (NC_044942.1) | MN394630.3 |
|  |  |  |  | NC_044944.1 | Unknown(NC_044943.1) |  |
|  |  |  |  | NC_044946.1[T] | Unknown(MZ945537.1) |  |
|  |  |  |  | PP750552.1[T] |  |  |
|  |  |  |  | OZ005801.1[T] |  |  |
|  |  |  |  | OZ003747.1[T] |  |  |
|  |  |  |  | ON409980.1[T] |  |  |
|  |  |  |  | MZ566623.1[T] |  |  |
|  |  |  |  | MT956648.1[T] |  |  |
|  |  |  |  | MH025918.1[T] |  |  |
|  |  |  |  |  |  |  |
| 93527 | 144283* | 67808 | 111914* | ^NC_044951.1 | Unknown (PP592890.1) | PP828951.1 |
|  |  |  |  | NC_044950.1 | Unknown(NC_044941.1) |  |
|  |  |  |  | NC_044952.1 | Unknown(NC_044943.1) |  |
|  |  |  |  |  | Unknown(NC_044955.1) |  |
|  |  |  |  |  | Unknown(NC_044956.1) |  |
|  |  |  |  |  | Unknown(NC_001659.2) |  |
|  |  |  |  |  | Unknown(OR371517.1) |  |
|  |  |  |  |  | Unknown(OR387520.1) |  |
|  |  |  |  |  | Unknown(MZ945536.1) |  |
|  |  |  |  |  | Unknown(MZ945537.1) |  |
|  |  |  |  |  | Unknown(MW736602.1) |  |
|  |  |  |  |  | Unknown(MN270972.1) |  |
|  |  |  |  |  | Unknown(MN270973.1) |  |
|  |  |  |  |  |  |  |
| 88147 | 88329* | 67636 | 67818* | ^MN336500.3 | Unknown (PP828951.1) | OR449224.1 |
|  |  |  |  | MN630494.2 | Unknown(NC_044953.1) |  |
|  |  |  |  | MN641876.2 |  |  |
|  |  |  |  | MN641877.2 |  |  |
|  |  |  |  | MN394630.3[T] |  |  |
|  |  |  |  |  |  |  |
| 16610* | 31157 | 11623* | 20547 | ^OR420801.1 | MN394630.3 | Unknown (OR449224.1) |
|  |  |  |  | OM249788.1 |  | Unknown(PP529961.1) |
|  |  |  |  | MZ202520.1 |  | Unknown(PP355086.1) |
|  |  |  |  |  |  | Unknown(ON409983.1) |
|  |  |  |  |  |  | Unknown(OF448913.1) |
|  |  |  |  |  |  | Unknown(OM105586.1) |
|  |  |  |  |  |  | Unknown(ON380540.1) |
|  |  |  |  |  |  | Unknown(ON400500.1) |
|  |  |  |  |  |  | Unknown(OL310288.1) |
|  |  |  |  |  |  |  |
| 92802 | 94556 | 71815 | 73298 | ^OR420801.1 | NC_044946.1 | NC_044942.1 |
|  |  |  |  |  | OZ005801.1 | NC_044941.1 |
|  |  |  |  |  | OZ003747.1 | NC_044943.1 |
|  |  |  |  |  | ON409980.1 | NC_044955.1 |
|  |  |  |  |  | MZ566623.1 | NC_044956.1 |
|  |  |  |  |  | MH025918.1 | NC_001659.2 |
|  |  |  |  |  |  | OR371517.1 |
|  |  |  |  |  |  | PP592890.1 |
|  |  |  |  |  |  | OR387520.1 |
|  |  |  |  |  |  | MZ945536.1 |
|  |  |  |  |  |  | MZ945537.1 |
|  |  |  |  |  |  | MW736602.1 |
|  |  |  |  |  |  | LR881473.1 |
|  |  |  |  |  |  | MN270972.1 |
|  |  |  |  |  |  | MN270973.1 |
|  |  |  |  |  |  |  |
| 70227 | 88329* | 48464 | 65622* | ^MN318203.3 | Unknown (PP592890.1) | NC_044949.1 |
|  |  |  |  | MN336500.3[P] | Unknown(NC_044941.1) | NC_044950.1 |
|  |  |  |  | MN394630.3[P] | Unknown(NC_044942.1) | NC_044952.1 |
|  |  |  |  |  | Unknown(NC_044943.1) |  |
|  |  |  |  |  | Unknown(NC_044955.1) |  |
|  |  |  |  |  | Unknown(NC_044956.1) |  |
|  |  |  |  |  | Unknown(PP810980.1) |  |
|  |  |  |  |  | Unknown(OR371517.1) |  |
|  |  |  |  |  | Unknown(PP348677.1) |  |
|  |  |  |  |  | Unknown(OQ504954.1) |  |
|  |  |  |  |  | Unknown(OQ504956.1) |  |
|  |  |  |  |  | Unknown(PP478517.1) |  |
|  |  |  |  |  | Unknown(OR387520.1) |  |
|  |  |  |  |  | Unknown(MZ945536.1) |  |
|  |  |  |  |  | Unknown(MZ945537.1) |  |
|  |  |  |  |  | Unknown(MW736602.1) |  |
|  |  |  |  |  | Unknown(MN270972.1) |  |
|  |  |  |  |  | Unknown(MN270973.1) |  |
|  |  |  |  |  |  |  |
| 63177 | 65570 | 43597 | 45940 | ^MZ566623.1 | Unknown (NC_044945.1) | OZ005801.1 |
|  |  |  |  |  | Unknown(NC_044946.1) | NC_044944.1 |
|  |  |  |  |  | Unknown(OZ005801.1) | NC_044945.1 |
|  |  |  |  |  | Unknown(OZ003747.1) | PP750552.1 |
|  |  |  |  |  | Unknown(ON409980.1) | MT956648.1 |
|  |  |  |  |  | Unknown(MH025918.1) |  |
|  |  |  |  |  |  |  |
| 16462 | 20185 | 12497 | 14100 | NC_044952.1 | Unknown (NC_044944.1) | NC_044950.1 |
|  |  |  |  |  | Unknown(NC_044946.1) | NC_044943.1 |
|  |  |  |  |  | Unknown(PP750552.1) | PP810980.1 |
|  |  |  |  |  | Unknown(OZ005801.1) | PP828951.1 |
|  |  |  |  |  | Unknown(OZ003747.1) | PP348677.1 |
|  |  |  |  |  | Unknown(ON409980.1) | OQ504954.1 |
|  |  |  |  |  | Unknown(MZ566623.1) | OQ504955.1 |
|  |  |  |  |  | Unknown(MH025918.1) | OQ504956.1 |
|  |  |  |  |  |  | PP478517.1 |
|  |  |  |  |  |  | MZ945536.1 |
|  |  |  |  |  |  | MZ945537.1 |
|  |  |  |  |  |  |  |
| 25614* | 28121 | 8457* | 8881 | NC_044942.1 | Unknown (PP355086.1) | OR371517.1 |
|  |  |  |  |  | Unknown(NC_044951.1) | NC_044941.1 |
|  |  |  |  |  | Unknown(PP529961.1) | NC_044955.1 |
|  |  |  |  |  | Unknown(ON409983.1) | NC_044956.1 |
|  |  |  |  |  | Unknown(OF448913.1) | NC_001659.2 |
|  |  |  |  |  | Unknown(OM105586.1) | PP592890.1 |
|  |  |  |  |  | Unknown(ON380540.1) | OR387520.1 |
|  |  |  |  |  | Unknown(OL310288.1) | MW736602.1 |
|  |  |  |  |  |  | MN270972.1 |
|  |  |  |  |  |  | MN270973.1 |
|  |  |  |  |  |  |  |
| 1771 | 3206 | 709 | 1467 | ^OK358852.1 | Unknown (NC_044953.1) | ON963982.2 |
|  |  |  |  | OR227304.1 |  | NC_044948.1 |
|  |  |  |  |  |  | OR660089.1 |
|  |  |  |  |  |  | PP529961.1 |
|  |  |  |  |  |  | OR290104.2 |
|  |  |  |  |  |  | OF448913.1 |
|  |  |  |  |  |  | OM105586.1 |
|  |  |  |  |  |  | ON380540.1 |
|  |  |  |  |  |  | OL310288.1 |
|  |  |  |  |  |  | MW656282.1 |
|  |  |  |  |  |  |  |
| 92969* | 93526* | 72112* | 72556* | NC_044950.1 | Unknown (PP750552.1) | ON380539.1 |
|  |  |  |  | NC_044952.1[P] | Unknown(MT956648.1) | NC_044948.1 |
|  |  |  |  |  |  | OR660089.1 |
|  |  |  |  |  |  | PP529961.1 |
|  |  |  |  |  |  | PP355086.1 |
|  |  |  |  |  |  | OP479889.1 |
|  |  |  |  |  |  | OR449224.1 |
|  |  |  |  |  |  | OR290104.2 |
|  |  |  |  |  |  | OR227304.1 |
|  |  |  |  |  |  | OR180113.1 |
|  |  |  |  |  |  | OK358852.1 |
|  |  |  |  |  |  | OP672342.1 |
|  |  |  |  |  |  | ON409979.1 |
|  |  |  |  |  |  | ON963982.2 |
|  |  |  |  |  |  | MW656282.1 |
|  |  |  |  |  |  |  |
| 48065* | 145331 | 35180* | 119423 | ^MN641877.2 | NC_044950.1 | MN630494.2 |
|  |  |  |  | MN394630.3 | NC_044951.1 |  |
|  |  |  |  |  | NC_044953.1 |  |
|  |  |  |  |  | NC_001659.2 |  |
|  |  |  |  |  |  |  |
| 144431* | 145122 | 116832* | 117452 | NC_044950.1 | Unknown (MN336500.3) | PP355086.1 |
|  |  |  |  | NC_044951.1 |  | NC_044948.1 |
|  |  |  |  |  |  | NC_044952.1 |
|  |  |  |  |  |  | OR660089.1 |
|  |  |  |  |  |  | PP529961.1 |
|  |  |  |  |  |  | OP479889.1 |
|  |  |  |  |  |  | OP718535.1 |
|  |  |  |  |  |  | OR449224.1 |
|  |  |  |  |  |  | OR290104.2 |
|  |  |  |  |  |  | OR227304.1 |
|  |  |  |  |  |  | OR180113.1 |
|  |  |  |  |  |  | OK358852.1 |
|  |  |  |  |  |  | OP672342.1 |
|  |  |  |  |  |  | ON409979.1 |
|  |  |  |  |  |  | ON409983.1 |
|  |  |  |  |  |  | OF448913.1 |
|  |  |  |  |  |  | OM105586.1 |
|  |  |  |  |  |  | ON380539.1 |
|  |  |  |  |  |  | ON380540.1 |
|  |  |  |  |  |  | ON400500.1 |
|  |  |  |  |  |  | ON963982.2 |
|  |  |  |  |  |  | OL310288.1 |
|  |  |  |  |  |  | MW521382.1 |
|  |  |  |  |  |  | MW656282.1 |
|  |  |  |  |  |  |  |
| 92831* | 93526* | 67223* | 67807* | ^NC_044951.1 | PP750552.1 | OP479889.1 |
|  |  |  |  |  |  | NC_044948.1 |
|  |  |  |  |  |  | OR660089.1 |
|  |  |  |  |  |  | PP529961.1 |
|  |  |  |  |  |  | PP355086.1 |
|  |  |  |  |  |  | OP718535.1 |
|  |  |  |  |  |  | OR449224.1 |
|  |  |  |  |  |  | OR290104.2 |
|  |  |  |  |  |  | OR227304.1 |
|  |  |  |  |  |  | OR180113.1 |
|  |  |  |  |  |  | OK358852.1 |
|  |  |  |  |  |  | OP672342.1 |
|  |  |  |  |  |  | ON409979.1 |
|  |  |  |  |  |  | ON380539.1 |
|  |  |  |  |  |  | ON963982.2 |
|  |  |  |  |  |  | MW656282.1 |
|  |  |  |  |  |  |  |
| 92807* | 92968* | 70449* | 70604* | ^PP750552.1 | NC_044950.1 | Unknown (MW521382.1) |
|  |  |  |  | NC_044945.1[P] | NC_044941.1 | Unknown(OR660089.1) |
|  |  |  |  | NC_044946.1[P] | NC_044942.1 | Unknown(PP529961.1) |
|  |  |  |  | OZ005801.1[P] | NC_044953.1 | Unknown(PP355086.1) |
|  |  |  |  | OZ003747.1[P] | NC_044955.1 | Unknown(OP479889.1) |
|  |  |  |  | ON409980.1[P] | NC_044956.1 | Unknown(OR449224.1) |
|  |  |  |  | MZ566623.1[P] | NC_001659.2 | Unknown(OR227304.1) |
|  |  |  |  | MT956648.1 | OR371517.1 | Unknown(OK358852.1) |
|  |  |  |  | MH025918.1[P] | PP592890.1 | Unknown(OP672342.1) |
|  |  |  |  |  | OR387520.1 | Unknown(ON409979.1) |
|  |  |  |  |  | MW736602.1 | Unknown(ON380539.1) |
|  |  |  |  |  | MN270972.1 | Unknown(ON400500.1) |
|  |  |  |  |  | MN270973.1 | Unknown(ON963982.2) |
|  |  |  |  |  |  | Unknown(MW656282.1) |
|  |  |  |  |  |  |  |
| 16610* | 20222 | 11513* | 14012 | ^MN394630.3 | NC_044946.1 | MN641877.2 |
|  |  |  |  | NC_044941.1[P] | NC_044944.1 |  |
|  |  |  |  | NC_044943.1 | PP750552.1 |  |
|  |  |  |  | NC_044950.1 | OZ005801.1 |  |
|  |  |  |  | NC_044953.1 | OZ003747.1 |  |
|  |  |  |  | NC_044955.1[P] | ON409980.1 |  |
|  |  |  |  | NC_044956.1[P] | MZ566623.1 |  |
|  |  |  |  | PP810980.1 | MH025918.1 |  |
|  |  |  |  | PP828951.1 |  |  |
|  |  |  |  | OR371517.1[P] |  |  |
|  |  |  |  | PP348677.1 |  |  |
|  |  |  |  | OQ504954.1 |  |  |
|  |  |  |  | OQ504955.1 |  |  |
|  |  |  |  | OQ504956.1 |  |  |
|  |  |  |  | PP592890.1[P] |  |  |
|  |  |  |  | PP478517.1 |  |  |
|  |  |  |  | OR387520.1[P] |  |  |
|  |  |  |  | MZ945536.1 |  |  |
|  |  |  |  | MZ945537.1 |  |  |
|  |  |  |  | MW736602.1[P] |  |  |
|  |  |  |  | MN270972.1[P] |  |  |
|  |  |  |  | MN270973.1[P] |  |  |
|  |  |  |  | MN336500.3 |  |  |
|  |  |  |  |  |  |  |
| 44656 | 55848* | 32088 | 42678* | ^MN641877.2 | NC_044952.1 | NC_044945.1 |
|  |  |  |  | NC_044948.1[T] |  |  |
|  |  |  |  | NC_044952.1[T] |  |  |
|  |  |  |  |  |  |  |
| 39303 | 48079* | 24052 | 32173* | ^NC_044949.1 | Unknown (NC_044941.1) | PP828951.1 |
|  |  |  |  | NC_044950.1[P] | Unknown(NC_044942.1) | NC_044953.1 |
|  |  |  |  | NC_044952.1 | Unknown(NC_044955.1) |  |
|  |  |  |  |  | Unknown(NC_044956.1) |  |
|  |  |  |  |  | Unknown(OR371517.1) |  |
|  |  |  |  |  | Unknown(PP592890.1) |  |
|  |  |  |  |  | Unknown(OR387520.1) |  |
|  |  |  |  |  | Unknown(MW736602.1) |  |
|  |  |  |  |  | Unknown(MN270972.1) |  |
|  |  |  |  |  | Unknown(MN270973.1) |  |
|  |  |  |  |  |  |  |
| 8152 | 16604* | 4451 | 10918* | ^NC_044949.1 | OM249788.1 | MH025918.1 |
|  |  |  |  | NC_044948.1[P] | MZ202520.1 | NC_044944.1 |
|  |  |  |  | NC_044950.1 |  | NC_044946.1 |
|  |  |  |  | NC_044952.1 |  | PP750552.1 |
|  |  |  |  | OR660089.1[P] |  | OZ005801.1 |
|  |  |  |  | PP529961.1 |  | OZ003747.1 |
|  |  |  |  | PP355086.1[P] |  | ON409980.1 |
|  |  |  |  | OP479889.1[P] |  | MZ566623.1 |
|  |  |  |  | OP718535.1[P] |  |  |
|  |  |  |  | OR449224.1[P] |  |  |
|  |  |  |  | OR290104.2[P] |  |  |
|  |  |  |  | OR227304.1[P] |  |  |
|  |  |  |  | OR180113.1[P] |  |  |
|  |  |  |  | OK358852.1[P] |  |  |
|  |  |  |  | OP672342.1[P] |  |  |
|  |  |  |  | ON409979.1[P] |  |  |
|  |  |  |  | ON409983.1 |  |  |
|  |  |  |  | OF448913.1 |  |  |
|  |  |  |  | OM105586.1 |  |  |
|  |  |  |  | ON380539.1[P] |  |  |
|  |  |  |  | ON380540.1 |  |  |
|  |  |  |  | ON400500.1[P] |  |  |
|  |  |  |  | ON963982.2[P] |  |  |
|  |  |  |  | OL310288.1 |  |  |
|  |  |  |  | MW521382.1[P] |  |  |
|  |  |  |  | MW656282.1[P] |  |  |
|  |  |  |  |  |  |  |
| 66496 | 89977* | 42547 | 64737* | NC_044951.1 | OR660089.1 | Unknown (PP348677.1) |
|  |  |  |  |  | NC_044948.1 | Unknown(LR881473.1) |
|  |  |  |  |  | PP529961.1 |  |
|  |  |  |  |  | PP355086.1 |  |
|  |  |  |  |  | OP479889.1 |  |
|  |  |  |  |  | OP718535.1 |  |
|  |  |  |  |  | OR449224.1 |  |
|  |  |  |  |  | OR290104.2 |  |
|  |  |  |  |  | OR227304.1 |  |
|  |  |  |  |  | OR180113.1 |  |
|  |  |  |  |  | OK358852.1 |  |
|  |  |  |  |  | OP672342.1 |  |
|  |  |  |  |  | ON409979.1 |  |
|  |  |  |  |  | ON409983.1 |  |
|  |  |  |  |  | OF448913.1 |  |
|  |  |  |  |  | OM105586.1 |  |
|  |  |  |  |  | ON380539.1 |  |
|  |  |  |  |  | ON380540.1 |  |
|  |  |  |  |  | ON400500.1 |  |
|  |  |  |  |  | ON963982.2 |  |
|  |  |  |  |  | OL310288.1 |  |
|  |  |  |  |  | MW521382.1 |  |
|  |  |  |  |  | MW656282.1 |  |
|  |  |  |  |  |  |  |
| 7634 | 16604* | 5721 | 13286* | ^NC_044953.1 | NC_044945.1 | MZ945536.1 |
|  |  |  |  |  | NC_044944.1 | NC_044943.1 |
|  |  |  |  |  | NC_044946.1 | PP810980.1 |
|  |  |  |  |  | PP750552.1 | PP348677.1 |
|  |  |  |  |  | OZ005801.1 | OQ504954.1 |
|  |  |  |  |  | OZ003747.1 | OQ504956.1 |
|  |  |  |  |  | ON409980.1 | PP478517.1 |
|  |  |  |  |  | MZ566623.1 | MZ945537.1 |
|  |  |  |  |  |  |  |
| 92970* | 93041* | 66641* | 66712* | ^MT956648.1 | NC_001659.2 | NC_044944.1 |
|  |  |  |  | NC_044945.1 | NC_044941.1 |  |
|  |  |  |  | NC_044946.1[T] | NC_044942.1 |  |
|  |  |  |  | PP750552.1 | NC_044943.1 |  |
|  |  |  |  | OZ003747.1[T] | MZ945536.1 |  |
|  |  |  |  | ON409980.1[T] | MZ945537.1 |  |
|  |  |  |  |  | LR881473.1 |  |
|  |  |  |  |  |  |  |
| 4302 | 6906 | 3077 | 5339 | NC_044945.1 | Unknown (MZ566623.1) | NC_044944.1 |
|  |  |  |  |  | Unknown(NC_044944.1) | NC_044946.1 |
|  |  |  |  |  | Unknown(PP750552.1) | OZ005801.1 |
|  |  |  |  |  | Unknown(MT956648.1) | OZ003747.1 |
|  |  |  |  |  |  | ON409980.1 |
|  |  |  |  |  |  | MZ566623.1 |
|  |  |  |  |  |  | MH025918.1 |
|  |  |  |  |  |  |  |
| 144411 | 145261 | 118609 | 119382 | ^NC_044945.1 | NC_001659.2 | MW521382.1 |
|  |  |  |  | NC_044944.1 | LR881473.1 | NC_044948.1 |
|  |  |  |  | PP750552.1 |  | OR660089.1 |
|  |  |  |  | MT956648.1 |  | PP529961.1 |
|  |  |  |  |  |  | PP355086.1 |
|  |  |  |  |  |  | OP479889.1 |
|  |  |  |  |  |  | OP718535.1 |
|  |  |  |  |  |  | OR449224.1 |
|  |  |  |  |  |  | OR290104.2 |
|  |  |  |  |  |  | OR227304.1 |
|  |  |  |  |  |  | OR180113.1 |
|  |  |  |  |  |  | OK358852.1 |
|  |  |  |  |  |  | OP672342.1 |
|  |  |  |  |  |  | ON409979.1 |
|  |  |  |  |  |  | ON409983.1 |
|  |  |  |  |  |  | OF448913.1 |
|  |  |  |  |  |  | OM105586.1 |
|  |  |  |  |  |  | ON380539.1 |
|  |  |  |  |  |  | ON380540.1 |
|  |  |  |  |  |  | ON400500.1 |
|  |  |  |  |  |  | ON963982.2 |
|  |  |  |  |  |  | OL310288.1 |
|  |  |  |  |  |  | MW656282.1 |
|  |  |  |  |  |  |  |
| 51209 | 52239 | 38251 | 39189 | ^MN641877.2 | NC_044952.1 | MN630494.2 |
|  |  |  |  |  |  |  |
| 92970* | 93041* | 69206* | 69277* | ^OZ005801.1 | NC_044941.1 | NC_044944.1 |
|  |  |  |  | MZ566623.1 | NC_044942.1 |  |
|  |  |  |  | MH025918.1 | NC_044955.1 |  |
|  |  |  |  |  | NC_044956.1 |  |
|  |  |  |  |  | OR371517.1 |  |
|  |  |  |  |  | PP592890.1 |  |
|  |  |  |  |  | OR387520.1 |  |
|  |  |  |  |  | MW736602.1 |  |
|  |  |  |  |  | MN270972.1 |  |
|  |  |  |  |  | MN270973.1 |  |
|  |  |  |  |  |  |  |
| 49375 | 61946 | 34549 | 43274 | ^MN318203.3 | PP529961.1 | Unknown (MZ566623.1) |
|  |  |  |  |  | NC_044952.1 | Unknown(NC_044944.1) |
|  |  |  |  |  | OQ504955.1 | Unknown(NC_044945.1) |
|  |  |  |  |  | PP355086.1 | Unknown(NC_044946.1) |
|  |  |  |  |  | OR449224.1 | Unknown(PP750552.1) |
|  |  |  |  |  | ON409983.1 | Unknown(OZ005801.1) |
|  |  |  |  |  | OF448913.1 | Unknown(OZ003747.1) |
|  |  |  |  |  | OM105586.1 | Unknown(ON409980.1) |
|  |  |  |  |  | ON380540.1 | Unknown(MT956648.1) |
|  |  |  |  |  | ON400500.1 | Unknown(MH025918.1) |
|  |  |  |  |  | OL310288.1 |  |
|  |  |  |  |  |  |  |
| 144431* | 145103 | 99367* | 99968 | NC_001659.2 | Unknown (MW521382.1) | OZ003747.1 |
|  |  |  |  | LR881473.1 | Unknown(NC_044948.1) | NC_044946.1 |
|  |  |  |  |  | Unknown(OR660089.1) | OZ005801.1 |
|  |  |  |  |  | Unknown(PP529961.1) | ON409980.1 |
|  |  |  |  |  | Unknown(PP355086.1) | MZ566623.1 |
|  |  |  |  |  | Unknown(OP479889.1) | MH025918.1 |
|  |  |  |  |  | Unknown(OP718535.1) |  |
|  |  |  |  |  | Unknown(OR449224.1) |  |
|  |  |  |  |  | Unknown(OR290104.2) |  |
|  |  |  |  |  | Unknown(OR227304.1) |  |
|  |  |  |  |  | Unknown(OR180113.1) |  |
|  |  |  |  |  | Unknown(OK358852.1) |  |
|  |  |  |  |  | Unknown(OP672342.1) |  |
|  |  |  |  |  | Unknown(ON409979.1) |  |
|  |  |  |  |  | Unknown(ON409983.1) |  |
|  |  |  |  |  | Unknown(OF448913.1) |  |
|  |  |  |  |  | Unknown(OM105586.1) |  |
|  |  |  |  |  | Unknown(ON380539.1) |  |
|  |  |  |  |  | Unknown(ON380540.1) |  |
|  |  |  |  |  | Unknown(ON400500.1) |  |
|  |  |  |  |  | Unknown(ON963982.2) |  |
|  |  |  |  |  | Unknown(OL310288.1) |  |
|  |  |  |  |  | Unknown(MW656282.1) |  |
|  |  |  |  |  |  |  |
| 44656 | 48047* | 36628 | 39920* | ^NC_044944.1 | Unknown (NC_001659.2) | MN318203.3 |
|  |  |  |  | NC_044945.1 |  |  |
|  |  |  |  | NC_044946.1 |  |  |
|  |  |  |  | PP750552.1 |  |  |
|  |  |  |  | OZ005801.1 |  |  |
|  |  |  |  | OZ003747.1 |  |  |
|  |  |  |  | ON409980.1 |  |  |
|  |  |  |  | MZ566623.1 |  |  |
|  |  |  |  | MT956648.1 |  |  |
|  |  |  |  | MH025918.1 |  |  |
|  |  |  |  |  |  |  |
| 226888 | 227544 | 180385 | 181022 | OP672342.1 | Unknown (NC_044950.1) | OP479889.1 |
|  |  |  |  |  | Unknown(NC_044949.1) | NC_044948.1 |
|  |  |  |  |  |  | OR660089.1 |
|  |  |  |  |  |  | PP529961.1 |
|  |  |  |  |  |  | PP355086.1 |
|  |  |  |  |  |  | OP718535.1 |
|  |  |  |  |  |  | OR449224.1 |
|  |  |  |  |  |  | OR290104.2 |
|  |  |  |  |  |  | OR227304.1 |
|  |  |  |  |  |  | OR180113.1 |
|  |  |  |  |  |  | OK358852.1 |
|  |  |  |  |  |  | OF448913.1 |
|  |  |  |  |  |  | OM105586.1 |
|  |  |  |  |  |  | ON380539.1 |
|  |  |  |  |  |  | ON380540.1 |
|  |  |  |  |  |  | ON400500.1 |
|  |  |  |  |  |  | ON963982.2 |
|  |  |  |  |  |  | OL310288.1 |
|  |  |  |  |  |  | MW521382.1 |
|  |  |  |  |  |  | MW656282.1 |
|  |  |  |  |  |  |  |
| 38965 | 48056* | 24956 | 33235* | ^MN318203.3 | ON400500.1 | MZ202520.1 |
|  |  |  |  |  | PP355086.1 |  |
|  |  |  |  |  | OR449224.1 |  |
|  |  |  |  |  | ON409983.1 |  |
|  |  |  |  |  | OF448913.1 |  |
|  |  |  |  |  | OM105586.1 |  |
|  |  |  |  |  | ON380540.1 |  |
|  |  |  |  |  | OL310288.1 |  |
|  |  |  |  |  |  |  |
| 228889 | 229243* | 188187 | 188348* | OR660089.1 | Unknown (NC_044951.1) | MW521382.1 |
|  |  |  |  |  |  | PP529961.1 |
|  |  |  |  |  |  | PP355086.1 |
|  |  |  |  |  |  | OP479889.1 |
|  |  |  |  |  |  | OP718535.1 |
|  |  |  |  |  |  | OR449224.1 |
|  |  |  |  |  |  | OR227304.1 |
|  |  |  |  |  |  | OK358852.1 |
|  |  |  |  |  |  | ON380539.1 |
|  |  |  |  |  |  | ON400500.1 |
|  |  |  |  |  |  | ON963982.2 |
|  |  |  |  |  |  | MW656282.1 |
|  |  |  |  |  |  |  |
| 6907* | 7587 | 3620* | 4269 | ^NC_044954.1 | NC_044944.1 | Unknown (NC_044945.1) |
|  |  |  |  | PP107957.1 |  |  |
|  |  |  |  |  |  |  |
| 195851 | 39303* | 160349 | 25282* | ^MN641876.2 | MN336500.3 | MN630494.2 |
|  |  |  |  |  |  |  |
| 20481* | 21196 | 15960* | 16667 | NC_044953.1 | Unknown (PP107957.1) | MN270973.1 |
|  |  |  |  | PP828951.1[P] | Unknown(NC_044954.1) | NC_044941.1 |
|  |  |  |  |  |  | NC_044943.1 |
|  |  |  |  |  |  | NC_044955.1 |
|  |  |  |  |  |  | NC_044956.1 |
|  |  |  |  |  |  | PP810980.1 |
|  |  |  |  |  |  | OR371517.1 |
|  |  |  |  |  |  | PP348677.1 |
|  |  |  |  |  |  | OQ504954.1 |
|  |  |  |  |  |  | OQ504955.1 |
|  |  |  |  |  |  | OQ504956.1 |
|  |  |  |  |  |  | PP592890.1 |
|  |  |  |  |  |  | PP478517.1 |
|  |  |  |  |  |  | OR387520.1 |
|  |  |  |  |  |  | MZ945536.1 |
|  |  |  |  |  |  | MZ945537.1 |
|  |  |  |  |  |  | MW736602.1 |
|  |  |  |  |  |  | MN270972.1 |
|  |  |  |  |  |  |  |
| 228834* | 229243* | 182305* | 182591* | OP672342.1 | Unknown (NC_044951.1) | PP529961.1 |
|  |  |  |  |  |  | NC_044948.1 |
|  |  |  |  |  |  | PP355086.1 |
|  |  |  |  |  |  | OP479889.1 |
|  |  |  |  |  |  | OP718535.1 |
|  |  |  |  |  |  | OR449224.1 |
|  |  |  |  |  |  | OR290104.2 |
|  |  |  |  |  |  | OR227304.1 |
|  |  |  |  |  |  | OR180113.1 |
|  |  |  |  |  |  | OK358852.1 |
|  |  |  |  |  |  | OF448913.1 |
|  |  |  |  |  |  | OM105586.1 |
|  |  |  |  |  |  | ON380539.1 |
|  |  |  |  |  |  | ON380540.1 |
|  |  |  |  |  |  | ON400500.1 |
|  |  |  |  |  |  | ON963982.2 |
|  |  |  |  |  |  | OL310288.1 |
|  |  |  |  |  |  | MW521382.1 |
|  |  |  |  |  |  | MW656282.1 |
|  |  |  |  |  |  |  |
| 7302* | 7394* | 5735* | 5812* | NC_044945.1 | MZ202520.1 | OZ003747.1 |
|  |  |  |  |  | NC_044941.1 | NC_044946.1 |
|  |  |  |  |  | NC_044942.1 | OZ005801.1 |
|  |  |  |  |  | NC_044950.1 | ON409980.1 |
|  |  |  |  |  | OM249788.1 | MZ566623.1 |
|  |  |  |  |  | MW736602.1 | MH025918.1 |
|  |  |  |  |  | MN270972.1 |  |
|  |  |  |  |  | MN270973.1 |  |
|  |  |  |  |  |  |  |
| 7401* | 7508* | 5819* | 5926* | NC_044945.1 | Unknown (MW736602.1) | MZ566623.1 |
|  |  |  |  | PP750552.1[P] | Unknown(NC_044941.1) | NC_044944.1 |
|  |  |  |  | MT956648.1[P] | Unknown(NC_044955.1) | OZ005801.1 |
|  |  |  |  |  | Unknown(MN270973.1) |  |
|  |  |  |  |  |  |  |
| 7804* | 7828* | 2959* | 2983* | ^OZ003747.1 | MZ202520.1 | NC_044945.1 |
|  |  |  |  |  | NC_044941.1 | NC_044944.1 |
|  |  |  |  |  | NC_044949.1 |  |
|  |  |  |  |  | OM249788.1 |  |
|  |  |  |  |  |  |  |
| 94076 | 97099* | 72251 | 74873* | ^MZ202520.1 | LR881473.1 | MH025918.1 |
|  |  |  |  | OR420801.1[P] | NC_044941.1 | NC_044946.1 |
|  |  |  |  | OM249788.1 | NC_044942.1 | OZ005801.1 |
|  |  |  |  |  | NC_044955.1 | OZ003747.1 |
|  |  |  |  |  | NC_044956.1 | ON409980.1 |
|  |  |  |  |  | NC_001659.2 | MZ566623.1 |
|  |  |  |  |  | OR371517.1 |  |
|  |  |  |  |  | PP592890.1 |  |
|  |  |  |  |  | OR387520.1 |  |
|  |  |  |  |  | MZ945537.1 |  |
|  |  |  |  |  | MW736602.1 |  |
|  |  |  |  |  | MN270972.1 |  |
|  |  |  |  |  | MN270973.1 |  |
|  |  |  |  |  |  |  |
| 87421 | 92690* | 67529 | 72486* | ^NC_044952.1 | Unknown (NC_001659.2) | MZ202520.1 |
|  |  |  |  | NC_044949.1[P] | Unknown(NC_044941.1) | OM249788.1 |
|  |  |  |  |  | Unknown(NC_044942.1) |  |
|  |  |  |  |  | Unknown(NC_044955.1) |  |
|  |  |  |  |  | Unknown(NC_044956.1) |  |
|  |  |  |  |  | Unknown(OR371517.1) |  |
|  |  |  |  |  | Unknown(PP592890.1) |  |
|  |  |  |  |  | Unknown(OR387520.1) |  |
|  |  |  |  |  | Unknown(MZ945536.1) |  |
|  |  |  |  |  | Unknown(MZ945537.1) |  |
|  |  |  |  |  | Unknown(MW736602.1) |  |
|  |  |  |  |  | Unknown(LR881473.1) |  |
|  |  |  |  |  | Unknown(MN270972.1) |  |
|  |  |  |  |  | Unknown(MN270973.1) |  |
|  |  |  |  |  |  |  |
| 70051 | 87420* | 50949 | 67528* | ^NC_044952.1 | Unknown (NC_044943.1) | OR449224.1 |
|  |  |  |  | NC_044949.1[P] |  | NC_044948.1 |
|  |  |  |  |  |  | OR660089.1 |
|  |  |  |  |  |  | PP529961.1 |
|  |  |  |  |  |  | PP355086.1 |
|  |  |  |  |  |  | OP479889.1 |
|  |  |  |  |  |  | OP718535.1 |
|  |  |  |  |  |  | OR290104.2 |
|  |  |  |  |  |  | OR227304.1 |
|  |  |  |  |  |  | OR180113.1 |
|  |  |  |  |  |  | OK358852.1 |
|  |  |  |  |  |  | OP672342.1 |
|  |  |  |  |  |  | ON409979.1 |
|  |  |  |  |  |  | ON409983.1 |
|  |  |  |  |  |  | OF448913.1 |
|  |  |  |  |  |  | OM105586.1 |
|  |  |  |  |  |  | ON380539.1 |
|  |  |  |  |  |  | ON380540.1 |
|  |  |  |  |  |  | ON963982.2 |
|  |  |  |  |  |  | OL310288.1 |
|  |  |  |  |  |  | MW521382.1 |
|  |  |  |  |  |  | MW656282.1 |
|  |  |  |  |  |  |  |
| 218788* | 225972* | 183913* | 189337* | NC_044945.1 | Unknown (NC_001659.2) | ON963982.2 |
|  |  |  |  | NC_044944.1 |  | OR660089.1 |
|  |  |  |  | NC_044946.1[P] |  | PP529961.1 |
|  |  |  |  | PP750552.1[P] |  | PP355086.1 |
|  |  |  |  | OZ005801.1[P] |  | OP479889.1 |
|  |  |  |  | OZ003747.1[P] |  | OR449224.1 |
|  |  |  |  | ON409980.1[P] |  | OR227304.1 |
|  |  |  |  | MZ566623.1[P] |  | OK358852.1 |
|  |  |  |  | MT956648.1[P] |  | OP672342.1 |
|  |  |  |  | MH025918.1[P] |  | ON409979.1 |
|  |  |  |  |  |  | ON380539.1 |
|  |  |  |  |  |  | ON400500.1 |
|  |  |  |  |  |  | MW521382.1 |
|  |  |  |  |  |  | MW656282.1 |
|  |  |  |  |  |  |  |
| 25716 | 28120 | 17248 | 18957 | ^MN641877.2 | Unknown (MN394630.3) | NC_044950.1 |
|  |  |  |  |  |  |  |
| 7893* | 21024 | 3995* | 12199 | ^ON409981.1 | MN270973.1 | NC_044952.1 |
|  |  |  |  |  | NC_044948.1 |  |
|  |  |  |  |  | OR660089.1 |  |
|  |  |  |  |  | PP529961.1 |  |
|  |  |  |  |  | PP355086.1 |  |
|  |  |  |  |  | OR290104.2 |  |
|  |  |  |  |  | OR227304.1 |  |
|  |  |  |  |  | OR180113.1 |  |
|  |  |  |  |  | OK358852.1 |  |
|  |  |  |  |  | ON409979.1 |  |
|  |  |  |  |  | ON409983.1 |  |
|  |  |  |  |  | OF448913.1 |  |
|  |  |  |  |  | OM105586.1 |  |
|  |  |  |  |  | ON380539.1 |  |
|  |  |  |  |  | ON380540.1 |  |
|  |  |  |  |  | ON963982.2 |  |
|  |  |  |  |  | OL310288.1 |  |
|  |  |  |  |  | MW521382.1 |  |
|  |  |  |  |  | MW656282.1 |  |
|  |  |  |  |  |  |  |
| 92820 | 93049 | 71311 | 71525 | MZ202520.1 | OR449224.1 | NC_044942.1 |
|  |  |  |  | OM249788.1 | OR660089.1 | NC_044941.1 |
|  |  |  |  |  | PP355086.1 | NC_044943.1 |
|  |  |  |  |  | OR290104.2 | NC_044955.1 |
|  |  |  |  |  | OR227304.1 | NC_044956.1 |
|  |  |  |  |  | OP672342.1 | NC_001659.2 |
|  |  |  |  |  | ON963982.2 | OR371517.1 |
|  |  |  |  |  | MW656282.1 | PP592890.1 |
|  |  |  |  |  |  | OR387520.1 |
|  |  |  |  |  |  | MZ945536.1 |
|  |  |  |  |  |  | MZ945537.1 |
|  |  |  |  |  |  | MW736602.1 |
|  |  |  |  |  |  | LR881473.1 |
|  |  |  |  |  |  | MN270972.1 |
|  |  |  |  |  |  | MN270973.1 |
|  |  |  |  |  |  |  |
| 19167 | 20558 | 16050 | 17339 | ^NC_044945.1 | Unknown (ON409980.1) | NC_044944.1 |
|  |  |  |  |  | Unknown(NC_044946.1) | PP750552.1 |
|  |  |  |  |  | Unknown(OZ005801.1) | MT956648.1 |
|  |  |  |  |  | Unknown(OZ003747.1) |  |
|  |  |  |  |  | Unknown(MZ566623.1) |  |
|  |  |  |  |  | Unknown(MH025918.1) |  |
|  |  |  |  |  |  |  |
| 20224* | 21060 | 11715* | 12542 | ^PP107957.1 | NC_044950.1 | NC_044952.1 |
|  |  |  |  | NC_044954.1 | NC_044941.1 |  |
|  |  |  |  |  | NC_044943.1 |  |
|  |  |  |  |  | NC_044955.1 |  |
|  |  |  |  |  | NC_044956.1 |  |
|  |  |  |  |  | PP810980.1 |  |
|  |  |  |  |  | OR371517.1 |  |
|  |  |  |  |  | PP348677.1 |  |
|  |  |  |  |  | OQ504954.1 |  |
|  |  |  |  |  | OQ504955.1 |  |
|  |  |  |  |  | OQ504956.1 |  |
|  |  |  |  |  | PP592890.1 |  |
|  |  |  |  |  | PP478517.1 |  |
|  |  |  |  |  | OR387520.1 |  |
|  |  |  |  |  | MZ945536.1 |  |
|  |  |  |  |  | MZ945537.1 |  |
|  |  |  |  |  | MW736602.1 |  |
|  |  |  |  |  | MN270972.1 |  |
|  |  |  |  |  | MN270973.1 |  |
|  |  |  |  |  |  |  |
| 29035 | 30965* | 19843 | 20521* | ^MN336500.3 | NC_044950.1 | MN394630.3 |
|  |  |  |  |  |  |  |
| 167495 | 177772* | 138373 | 146726* | NC_044952.1 | Unknown (MN318203.3) | MN630494.2 |
|  |  |  |  | NC_044951.1[P] |  | MN641876.2 |
|  |  |  |  |  |  | MN641877.2 |
|  |  |  |  |  |  |  |
| 124778 | 125430 | 95892 | 96079 | ^OR387520.1 | Unknown (MZ202520.1) | NC_001659.2 |
|  |  |  |  | NC_044956.1 |  | NC_044943.1 |
|  |  |  |  | OR371517.1[P] |  | MZ945537.1 |
|  |  |  |  |  |  |  |
| 231411* | 380 | 185303* | 2 | OQ504956.1 | Unknown (OK358852.1) | PP478517.1 |
|  |  |  |  |  |  |  |
| 36490 | 38185 | 19855 | 21399 | ^ON409981.1 | PP529961.1 | NC_044944.1 |
|  |  |  |  | PP107957.1 | NC_044941.1 | NC_044945.1 |
|  |  |  |  |  | NC_044942.1 | NC_044946.1 |
|  |  |  |  |  | NC_044953.1 | PP750552.1 |
|  |  |  |  |  | NC_044955.1 | OZ005801.1 |
|  |  |  |  |  | NC_044956.1 | OZ003747.1 |
|  |  |  |  |  | PP828951.1 | ON409980.1 |
|  |  |  |  |  | OR371517.1 | MZ566623.1 |
|  |  |  |  |  | OQ504955.1 | MT956648.1 |
|  |  |  |  |  | PP592890.1 | MH025918.1 |
|  |  |  |  |  | PP355086.1 |  |
|  |  |  |  |  | OR449224.1 |  |
|  |  |  |  |  | OR420801.1 |  |
|  |  |  |  |  | OR387520.1 |  |
|  |  |  |  |  | ON409983.1 |  |
|  |  |  |  |  | OF448913.1 |  |
|  |  |  |  |  | OM105586.1 |  |
|  |  |  |  |  | ON380540.1 |  |
|  |  |  |  |  | ON400500.1 |  |
|  |  |  |  |  | OL310288.1 |  |
|  |  |  |  |  | MW736602.1 |  |
|  |  |  |  |  | MZ202520.1 |  |
|  |  |  |  |  | MN270972.1 |  |
|  |  |  |  |  | MN270973.1 |  |
|  |  |  |  |  |  |  |
| 209086 | 209723 | 178483 | 179109 | ^NC_044944.1 | NC_044945.1 | PP750552.1 |
|  |  |  |  |  |  | MT956648.1 |
|  |  |  |  |  |  |  |
| 218788* | 223793 | 183278* | 185760 | ^LR881473.1 | NC_044953.1 | Unknown (OR449224.1) |
|  |  |  |  |  |  | Unknown(NC_044948.1) |
|  |  |  |  |  |  | Unknown(OR660089.1) |
|  |  |  |  |  |  | Unknown(PP529961.1) |
|  |  |  |  |  |  | Unknown(PP355086.1) |
|  |  |  |  |  |  | Unknown(OP479889.1) |
|  |  |  |  |  |  | Unknown(OP718535.1) |
|  |  |  |  |  |  | Unknown(OR290104.2) |
|  |  |  |  |  |  | Unknown(OR227304.1) |
|  |  |  |  |  |  | Unknown(OR180113.1) |
|  |  |  |  |  |  | Unknown(OK358852.1) |
|  |  |  |  |  |  | Unknown(OP672342.1) |
|  |  |  |  |  |  | Unknown(ON409979.1) |
|  |  |  |  |  |  | Unknown(ON409983.1) |
|  |  |  |  |  |  | Unknown(OF448913.1) |
|  |  |  |  |  |  | Unknown(OM105586.1) |
|  |  |  |  |  |  | Unknown(ON380539.1) |
|  |  |  |  |  |  | Unknown(ON380540.1) |
|  |  |  |  |  |  | Unknown(ON400500.1) |
|  |  |  |  |  |  | Unknown(ON963982.2) |
|  |  |  |  |  |  | Unknown(OL310288.1) |
|  |  |  |  |  |  | Unknown(MW521382.1) |
|  |  |  |  |  |  | Unknown(MW656282.1) |
|  |  |  |  |  |  |  |
| 93490 | 93853 | 71869 | 72064 | ^MZ202520.1 | NC_044945.1 | NC_044942.1 |
|  |  |  |  | OM249788.1 |  | NC_044941.1 |
|  |  |  |  |  |  | NC_044943.1 |
|  |  |  |  |  |  | NC_044955.1 |
|  |  |  |  |  |  | NC_044956.1 |
|  |  |  |  |  |  | NC_001659.2 |
|  |  |  |  |  |  | OR371517.1 |
|  |  |  |  |  |  | PP592890.1 |
|  |  |  |  |  |  | OR387520.1 |
|  |  |  |  |  |  | MZ945536.1 |
|  |  |  |  |  |  | MZ945537.1 |
|  |  |  |  |  |  | MW736602.1 |
|  |  |  |  |  |  | LR881473.1 |
|  |  |  |  |  |  | MN270972.1 |
|  |  |  |  |  |  | MN270973.1 |
|  |  |  |  |  |  |  |
| 48080* | 49145 | 30227* | 31287 | ^NC_044951.1 | MN641877.2 | PP529961.1 |
|  |  |  |  | NC_044949.1[P] | ON409981.1 | PP355086.1 |
|  |  |  |  | NC_044952.1 |  | ON409983.1 |
|  |  |  |  | MN630494.2[P] |  | OF448913.1 |
|  |  |  |  | MN641877.2[P] |  | OM105586.1 |
|  |  |  |  |  |  | ON380540.1 |
|  |  |  |  |  |  | OL310288.1 |
|  |  |  |  |  |  |  |
| 66508 | 67546* | 48594 | 49447* | ^LR881473.1 | MN318203.3 | Unknown (OZ003747.1) |
|  |  |  |  |  |  | Unknown(NC_044944.1) |
|  |  |  |  |  |  | Unknown(NC_044945.1) |
|  |  |  |  |  |  | Unknown(NC_044946.1) |
|  |  |  |  |  |  | Unknown(PP750552.1) |
|  |  |  |  |  |  | Unknown(OZ005801.1) |
|  |  |  |  |  |  | Unknown(ON409980.1) |
|  |  |  |  |  |  | Unknown(MZ566623.1) |
|  |  |  |  |  |  | Unknown(MT956648.1) |
|  |  |  |  |  |  | Unknown(MH025918.1) |
|  |  |  |  |  |  |  |
| 67414 | 67572* | 46654 | 46715* | ^MW361944.1 | PP355086.1 | Unknown (NC_044944.1) |
|  |  |  |  |  | NC_044948.1 | Unknown(NC_044945.1) |
|  |  |  |  |  | OR660089.1 | Unknown(NC_044946.1) |
|  |  |  |  |  | PP529961.1 | Unknown(PP750552.1) |
|  |  |  |  |  | OP479889.1 | Unknown(OZ005801.1) |
|  |  |  |  |  | OP718535.1 | Unknown(OZ003747.1) |
|  |  |  |  |  | OR449224.1 | Unknown(ON409980.1) |
|  |  |  |  |  | OR290104.2 | Unknown(MZ566623.1) |
|  |  |  |  |  | OR227304.1 | Unknown(MT956648.1) |
|  |  |  |  |  | OR180113.1 | Unknown(MH025918.1) |
|  |  |  |  |  | OK358852.1 |  |
|  |  |  |  |  | OP672342.1 |  |
|  |  |  |  |  | ON409979.1 |  |
|  |  |  |  |  | ON409983.1 |  |
|  |  |  |  |  | OF448913.1 |  |
|  |  |  |  |  | OM105586.1 |  |
|  |  |  |  |  | ON380539.1 |  |
|  |  |  |  |  | ON380540.1 |  |
|  |  |  |  |  | ON400500.1 |  |
|  |  |  |  |  | ON963982.2 |  |
|  |  |  |  |  | OL310288.1 |  |
|  |  |  |  |  | MW521382.1 |  |
|  |  |  |  |  | MW656282.1 |  |
|  |  |  |  |  |  |  |
| 145123* | 145566 | 112628* | 112997 | NC_044951.1 | LR881473.1 | PP529961.1 |
|  |  |  |  |  |  | NC_044948.1 |
|  |  |  |  |  |  | OR660089.1 |
|  |  |  |  |  |  | PP355086.1 |
|  |  |  |  |  |  | OP479889.1 |
|  |  |  |  |  |  | OP718535.1 |
|  |  |  |  |  |  | OR449224.1 |
|  |  |  |  |  |  | OR290104.2 |
|  |  |  |  |  |  | OR227304.1 |
|  |  |  |  |  |  | OR180113.1 |
|  |  |  |  |  |  | OK358852.1 |
|  |  |  |  |  |  | OP672342.1 |
|  |  |  |  |  |  | ON409979.1 |
|  |  |  |  |  |  | ON409983.1 |
|  |  |  |  |  |  | OF448913.1 |
|  |  |  |  |  |  | OM105586.1 |
|  |  |  |  |  |  | ON380539.1 |
|  |  |  |  |  |  | ON380540.1 |
|  |  |  |  |  |  | ON400500.1 |
|  |  |  |  |  |  | ON963982.2 |
|  |  |  |  |  |  | OL310288.1 |
|  |  |  |  |  |  | MW521382.1 |
|  |  |  |  |  |  | MW656282.1 |
|  |  |  |  |  |  |  |
| 21320* | 24582 | 15473* | 16552 | ^NC_044950.1 | MN336500.3 | MN641877.2 |
|  |  |  |  |  | MN394630.3 |  |
|  |  |  |  |  |  |  |
| 25718 | 28121 | 17148 | 18491 | ^MN394630.3 | NC_044950.1 | NC_044954.1 |
|  |  |  |  |  |  | NC_044941.1 |
|  |  |  |  |  |  | NC_044955.1 |
|  |  |  |  |  |  | NC_044956.1 |
|  |  |  |  |  |  | OR371517.1 |
|  |  |  |  |  |  | PP592890.1 |
|  |  |  |  |  |  | OR387520.1 |
|  |  |  |  |  |  | MW736602.1 |
|  |  |  |  |  |  | MN270972.1 |
|  |  |  |  |  |  | MN270973.1 |
|  |  |  |  |  |  |  |
| 25159 | 27963* | 17064 | 18797* | ^MN336500.3 | NC_044950.1 | PP107957.1 |
|  |  |  |  |  |  | NC_044941.1 |
|  |  |  |  |  |  | NC_044955.1 |
|  |  |  |  |  |  | NC_044956.1 |
|  |  |  |  |  |  | OR371517.1 |
|  |  |  |  |  |  | PP592890.1 |
|  |  |  |  |  |  | OR387520.1 |
|  |  |  |  |  |  | MW736602.1 |
|  |  |  |  |  |  | MN270972.1 |
|  |  |  |  |  |  | MN270973.1 |
|  |  |  |  |  |  |  |
| 56828 | 66054 | 38951 | 44250 | ^OZ005801.1 | Unknown (LR881473.1) | PP529961.1 |
|  |  |  |  | NC_044944.1 | Unknown(PP810980.1) | NC_044948.1 |
|  |  |  |  | NC_044945.1 | Unknown(PP348677.1) | OR660089.1 |
|  |  |  |  | NC_044946.1 | Unknown(OQ504954.1) | PP355086.1 |
|  |  |  |  | PP750552.1 | Unknown(OQ504956.1) | OP479889.1 |
|  |  |  |  | OZ003747.1 | Unknown(PP478517.1) | OP718535.1 |
|  |  |  |  | ON409980.1 |  | OR449224.1 |
|  |  |  |  | MZ566623.1 |  | OR290104.2 |
|  |  |  |  | MT956648.1 |  | OR227304.1 |
|  |  |  |  | MH025918.1 |  | OR180113.1 |
|  |  |  |  |  |  | OK358852.1 |
|  |  |  |  |  |  | OP672342.1 |
|  |  |  |  |  |  | ON409979.1 |
|  |  |  |  |  |  | ON409983.1 |
|  |  |  |  |  |  | OF448913.1 |
|  |  |  |  |  |  | OM105586.1 |
|  |  |  |  |  |  | ON380539.1 |
|  |  |  |  |  |  | ON380540.1 |
|  |  |  |  |  |  | ON400500.1 |
|  |  |  |  |  |  | ON963982.2 |
|  |  |  |  |  |  | OL310288.1 |
|  |  |  |  |  |  | MW521382.1 |
|  |  |  |  |  |  | MW656282.1 |
|  |  |  |  |  |  |  |
| 93071* | 97099* | 73703* | 77102* | ^NC_044945.1 | PP355086.1 | NC_044946.1 |
|  |  |  |  |  | OR660089.1 | OZ005801.1 |
|  |  |  |  |  | PP529961.1 | OZ003747.1 |
|  |  |  |  |  | OP479889.1 | ON409980.1 |
|  |  |  |  |  | OP718535.1 | MZ566623.1 |
|  |  |  |  |  | OR449224.1 | MH025918.1 |
|  |  |  |  |  | OR290104.2 |  |
|  |  |  |  |  | OR227304.1 |  |
|  |  |  |  |  | OR180113.1 |  |
|  |  |  |  |  | OK358852.1 |  |
|  |  |  |  |  | OP672342.1 |  |
|  |  |  |  |  | ON409979.1 |  |
|  |  |  |  |  | ON409983.1 |  |
|  |  |  |  |  | OF448913.1 |  |
|  |  |  |  |  | ON380539.1 |  |
|  |  |  |  |  | ON380540.1 |  |
|  |  |  |  |  | ON963982.2 |  |
|  |  |  |  |  | OL310288.1 |  |
|  |  |  |  |  | MW656282.1 |  |
|  |  |  |  |  |  |  |
| 164890* | 167420 | 135295* | 137571 | ^NC_044950.1 | MN394630.3 | Unknown (MN641876.2) |
|  |  |  |  |  | MN336500.3 |  |
|  |  |  |  |  |  |  |
| 76628 | 79816 | 54320 | 57339 | ^ON409979.1 | Unknown (PP828951.1) | NC_044941.1 |
|  |  |  |  | NC_044948.1 |  | NC_044942.1 |
|  |  |  |  | OR660089.1 |  | NC_044943.1 |
|  |  |  |  | PP529961.1 |  | NC_044955.1 |
|  |  |  |  | PP355086.1 |  | NC_044956.1 |
|  |  |  |  | OP479889.1 |  | NC_001659.2 |
|  |  |  |  | OP718535.1 |  | PP810980.1 |
|  |  |  |  | OR449224.1 |  | OR371517.1 |
|  |  |  |  | OR290104.2 |  | PP348677.1 |
|  |  |  |  | OR227304.1 |  | OQ504954.1 |
|  |  |  |  | OR180113.1 |  | OQ504955.1 |
|  |  |  |  | OK358852.1 |  | OQ504956.1 |
|  |  |  |  | OP672342.1 |  | PP592890.1 |
|  |  |  |  | ON409983.1 |  | PP478517.1 |
|  |  |  |  | OF448913.1 |  | OR420801.1 |
|  |  |  |  | OM105586.1 |  | OR387520.1 |
|  |  |  |  | ON380539.1 |  | OM249788.1 |
|  |  |  |  | ON380540.1 |  | MZ945536.1 |
|  |  |  |  | ON400500.1 |  | MZ945537.1 |
|  |  |  |  | ON963982.2 |  | MW736602.1 |
|  |  |  |  | OL310288.1 |  | MZ202520.1 |
|  |  |  |  | MW521382.1 |  | LR881473.1 |
|  |  |  |  | MW656282.1 |  | MN270972.1 |
|  |  |  |  |  |  | MN270973.1 |
|  |  |  |  |  |  |  |
| 48770* | 49619* | 31192* | 32036* | ^ON409981.1 | Unknown (MN641877.2) | PP529961.1 |
|  |  |  |  |  | Unknown(MN641876.2) | PP355086.1 |
|  |  |  |  |  |  | OR449224.1 |
|  |  |  |  |  |  |  |
| 48744* | 49622* | 40617* | 41490* | ^NC_044944.1 | Unknown (PP529961.1) | MN641877.2 |
|  |  |  |  | NC_044945.1 | Unknown(PP355086.1) |  |
|  |  |  |  | NC_044946.1 | Unknown(ON409983.1) |  |
|  |  |  |  | PP750552.1 | Unknown(OF448913.1) |  |
|  |  |  |  | OZ005801.1 | Unknown(OM105586.1) |  |
|  |  |  |  | OZ003747.1 | Unknown(ON380540.1) |  |
|  |  |  |  | ON409980.1 | Unknown(OL310288.1) |  |
|  |  |  |  | MZ566623.1 |  |  |
|  |  |  |  | MT956648.1 |  |  |
|  |  |  |  | MH025918.1 |  |  |
|  |  |  |  |  |  |  |
| 65042 | 67718 | 47210 | 48964 | NC_044952.1 | PP810980.1 | PP529961.1 |
|  |  |  |  |  | PP348677.1 | NC_044948.1 |
|  |  |  |  |  | OQ504954.1 | OR660089.1 |
|  |  |  |  |  | OQ504955.1 | PP355086.1 |
|  |  |  |  |  | OQ504956.1 | OP479889.1 |
|  |  |  |  |  | PP478517.1 | OP718535.1 |
|  |  |  |  |  |  | OR449224.1 |
|  |  |  |  |  |  | OR290104.2 |
|  |  |  |  |  |  | OR227304.1 |
|  |  |  |  |  |  | OR180113.1 |
|  |  |  |  |  |  | OK358852.1 |
|  |  |  |  |  |  | OP672342.1 |
|  |  |  |  |  |  | ON409979.1 |
|  |  |  |  |  |  | ON409983.1 |
|  |  |  |  |  |  | OF448913.1 |
|  |  |  |  |  |  | OM105586.1 |
|  |  |  |  |  |  | ON380539.1 |
|  |  |  |  |  |  | ON380540.1 |
|  |  |  |  |  |  | ON400500.1 |
|  |  |  |  |  |  | ON963982.2 |
|  |  |  |  |  |  | OL310288.1 |
|  |  |  |  |  |  | MW521382.1 |
|  |  |  |  |  |  | MW656282.1 |
|  |  |  |  |  |  |  |
| 39643 | 40415 | 30077 | 30845 | ^NC_044945.1 | MH025918.1 | Unknown (MT956648.1) |
|  |  |  |  |  | NC_044946.1 |  |
|  |  |  |  |  | OZ005801.1 |  |
|  |  |  |  |  | OZ003747.1 |  |
|  |  |  |  |  | ON409980.1 |  |
|  |  |  |  |  | MZ566623.1 |  |
|  |  |  |  |  |  |  |
| 146725 | 148579* | 120332 | 121862* | ^LR881473.1 | ON400500.1 | Unknown (ON409980.1) |
|  |  |  |  |  | OR660089.1 | Unknown(NC_044945.1) |
|  |  |  |  |  | PP529961.1 | Unknown(NC_044946.1) |
|  |  |  |  |  | PP355086.1 | Unknown(PP750552.1) |
|  |  |  |  |  | OP479889.1 | Unknown(OZ005801.1) |
|  |  |  |  |  | OR449224.1 | Unknown(OZ003747.1) |
|  |  |  |  |  | OR227304.1 | Unknown(MZ566623.1) |
|  |  |  |  |  | OK358852.1 | Unknown(MH025918.1) |
|  |  |  |  |  | OP672342.1 |  |
|  |  |  |  |  | ON409979.1 |  |
|  |  |  |  |  | OM105586.1 |  |
|  |  |  |  |  | ON380539.1 |  |
|  |  |  |  |  | ON963982.2 |  |
|  |  |  |  |  | MW521382.1 |  |
|  |  |  |  |  | MW656282.1 |  |
|  |  |  |  |  |  |  |
| 145106* | 145331 | 118946* | 119165 | LR881473.1 | Unknown (MN630494.2) | PP529961.1 |
|  |  |  |  | NC_001659.2 |  | NC_044948.1 |
|  |  |  |  |  |  | OR660089.1 |
|  |  |  |  |  |  | PP355086.1 |
|  |  |  |  |  |  | OP479889.1 |
|  |  |  |  |  |  | OP718535.1 |
|  |  |  |  |  |  | OR449224.1 |
|  |  |  |  |  |  | OR290104.2 |
|  |  |  |  |  |  | OR227304.1 |
|  |  |  |  |  |  | OR180113.1 |
|  |  |  |  |  |  | OK358852.1 |
|  |  |  |  |  |  | OP672342.1 |
|  |  |  |  |  |  | ON409979.1 |
|  |  |  |  |  |  | ON409983.1 |
|  |  |  |  |  |  | OF448913.1 |
|  |  |  |  |  |  | OM105586.1 |
|  |  |  |  |  |  | ON380539.1 |
|  |  |  |  |  |  | ON380540.1 |
|  |  |  |  |  |  | ON400500.1 |
|  |  |  |  |  |  | ON963982.2 |
|  |  |  |  |  |  | OL310288.1 |
|  |  |  |  |  |  | MW521382.1 |
|  |  |  |  |  |  | MW656282.1 |
|  |  |  |  |  |  |  |
| 211571 | 212953 | 177361 | 178564 | ^MN641877.2 | MN394630.3 | Unknown (MN336500.3) |
|  |  |  |  | MN630494.2 |  |  |
|  |  |  |  |  |  |  |
| 203537 | 208801 | 164284 | 168963 | ^MN318203.3 | MN394630.3 | Unknown (MN641877.2) |
|  |  |  |  |  | MN336500.3 | Unknown(MN630494.2) |
|  |  |  |  |  |  |  |
| 217900 | 217944 | 180804 | 180845 | ^MN394630.3 | Unknown (MN630494.2) | NC_044944.1 |
|  |  |  |  |  |  | NC_044945.1 |
|  |  |  |  |  |  | NC_044946.1 |
|  |  |  |  |  |  | PP750552.1 |
|  |  |  |  |  |  | OZ005801.1 |
|  |  |  |  |  |  | OZ003747.1 |
|  |  |  |  |  |  | ON409980.1 |
|  |  |  |  |  |  | MZ566623.1 |
|  |  |  |  |  |  | MT956648.1 |
|  |  |  |  |  |  | MH025918.1 |
|  |  |  |  |  |  |  |
| 8307 | 9318* | 7353 | 8254* | ^NC_044944.1 | NC_044954.1 | NC_044946.1 |
|  |  |  |  |  | PP107957.1 | OZ005801.1 |
|  |  |  |  |  |  | OZ003747.1 |
|  |  |  |  |  |  | ON409980.1 |
|  |  |  |  |  |  | MZ566623.1 |
|  |  |  |  |  |  | MH025918.1 |
|  |  |  |  |  |  |  |
| 36663 | 48047* | 25887 | 36555* | ^NC_044953.1 | OM249788.1 | Unknown (PP355086.1) |
|  |  |  |  |  | NC_044941.1 | Unknown(PP529961.1) |
|  |  |  |  |  | NC_044942.1 | Unknown(OR449224.1) |
|  |  |  |  |  | NC_044955.1 | Unknown(ON409983.1) |
|  |  |  |  |  | NC_044956.1 | Unknown(OF448913.1) |
|  |  |  |  |  | OR371517.1 | Unknown(OM105586.1) |
|  |  |  |  |  | PP592890.1 | Unknown(ON380540.1) |
|  |  |  |  |  | OR387520.1 | Unknown(ON400500.1) |
|  |  |  |  |  | MW736602.1 | Unknown(OL310288.1) |
|  |  |  |  |  | MZ202520.1 |  |
|  |  |  |  |  | MN270972.1 |  |
|  |  |  |  |  | MN270973.1 |  |
|  |  |  |  |  |  |  |
| 17763 | 18411 | 8166 | 8762 | MT956648.1 | Unknown (NC_044954.1) | ON409980.1 |
|  |  |  |  |  |  | NC_044946.1 |
|  |  |  |  |  |  | MH025918.1 |
|  |  |  |  |  |  |  |
| 211568 | 213070 | 170901 | 172221 | ^MN318203.3 | MN394630.3 | Unknown (MN336500.3) |
|  |  |  |  |  |  |  |
| 225334* | 225902* | 170171* | 170650* | ^OR449224.1 | Unknown (MZ945537.1) | NC_044942.1 |
|  |  |  |  |  |  | NC_044941.1 |
|  |  |  |  |  |  | NC_044955.1 |
|  |  |  |  |  |  | NC_044956.1 |
|  |  |  |  |  |  | NC_001659.2 |
|  |  |  |  |  |  | OR371517.1 |
|  |  |  |  |  |  | PP592890.1 |
|  |  |  |  |  |  | OR387520.1 |
|  |  |  |  |  |  | MW736602.1 |
|  |  |  |  |  |  | MN270972.1 |
|  |  |  |  |  |  | MN270973.1 |
|  |  |  |  |  |  |  |
| 41760 | 48053* | 24805 | 30477* | ^ON409981.1 | Unknown (PP828951.1) | NC_044941.1 |
|  |  |  |  | NC_044946.1[T] |  | NC_044942.1 |
|  |  |  |  | PP107957.1 |  | NC_044955.1 |
|  |  |  |  |  |  | NC_044956.1 |
|  |  |  |  |  |  | OR371517.1 |
|  |  |  |  |  |  | PP592890.1 |
|  |  |  |  |  |  | OR420801.1 |
|  |  |  |  |  |  | OR387520.1 |
|  |  |  |  |  |  | MW736602.1 |
|  |  |  |  |  |  | MZ202520.1 |
|  |  |  |  |  |  | MN270972.1 |
|  |  |  |  |  |  | MN270973.1 |
|  |  |  |  |  |  |  |
| 225333* | 225902* | 181072* | 181555* | ^OR371517.1 | Unknown (MZ945537.1) | PP529961.1 |
|  |  |  |  |  |  |  |
| 225333* | 225902* | 180909* | 181392* | ^NC_044956.1 | Unknown (MZ945537.1) | PP529961.1 |
|  |  |  |  |  |  |  |
| 20545 | 21123 | 14348 | 14921 | ^MN641877.2 | Unknown (PP348677.1) | MN394630.3 |
|  |  |  |  |  |  | MN336500.3 |
|  |  |  |  |  |  |  |
| 48746* | 49622* | 33379* | 34250* | ^PP107957.1 | Unknown (PP355086.1) | MN641877.2 |
|  |  |  |  |  | Unknown(PP529961.1) |  |
|  |  |  |  |  |  |  |
| 131778 | 131869 | 93104 | 93157 | ^NC_044943.1 | Unknown (PP828951.1) | OR371517.1 |
|  |  |  |  |  | Unknown(NC_044941.1) | PP828951.1 |
|  |  |  |  |  | Unknown(NC_044942.1) |  |
|  |  |  |  |  | Unknown(NC_044955.1) |  |
|  |  |  |  |  | Unknown(NC_044956.1) |  |
|  |  |  |  |  | Unknown(NC_001659.2) |  |
|  |  |  |  |  | Unknown(OR371517.1) |  |
|  |  |  |  |  | Unknown(PP592890.1) |  |
|  |  |  |  |  | Unknown(OR420801.1) |  |
|  |  |  |  |  | Unknown(OR387520.1) |  |
|  |  |  |  |  | Unknown(MW736602.1) |  |
|  |  |  |  |  | Unknown(MN270972.1) |  |
|  |  |  |  |  | Unknown(MN270973.1) |  |
|  |  |  |  |  |  |  |
| 225333* | 225894* | 171985* | 172047* | ^NC_044943.1 | OR387520.1 | Unknown (PP355086.1) |
|  |  |  |  |  | NC_044941.1 |  |
|  |  |  |  |  | NC_044942.1 |  |
|  |  |  |  |  | NC_001659.2 |  |
|  |  |  |  |  | PP592890.1 |  |
|  |  |  |  |  | OR420801.1 |  |
|  |  |  |  |  | OM249788.1 |  |
|  |  |  |  |  | MN270972.1 |  |
|  |  |  |  |  | MN270973.1 |  |
|  |  |  |  |  |  |  |
| 218201 | 218697* | 181268 | 181707* | ^MZ566623.1 | Unknown (MN318203.3) | PP750552.1 |
|  |  |  |  | NC_044946.1 |  | NC_044944.1 |
|  |  |  |  | OZ005801.1 |  | MT956648.1 |
|  |  |  |  | OZ003747.1 |  |  |
|  |  |  |  | ON409980.1 |  |  |
|  |  |  |  | MH025918.1 |  |  |
|  |  |  |  |  |  |  |
| 148123 | 148400 | 102530 | 102724 | ^NC_001659.2 | MN641876.2 | PP529961.1 |
|  |  |  |  |  |  | NC_044948.1 |
|  |  |  |  |  |  | OR660089.1 |
|  |  |  |  |  |  | PP355086.1 |
|  |  |  |  |  |  | OP479889.1 |
|  |  |  |  |  |  | OP718535.1 |
|  |  |  |  |  |  | OR449224.1 |
|  |  |  |  |  |  | OR290104.2 |
|  |  |  |  |  |  | OR227304.1 |
|  |  |  |  |  |  | OR180113.1 |
|  |  |  |  |  |  | OK358852.1 |
|  |  |  |  |  |  | OP672342.1 |
|  |  |  |  |  |  | ON409979.1 |
|  |  |  |  |  |  | ON409983.1 |
|  |  |  |  |  |  | OF448913.1 |
|  |  |  |  |  |  | OM105586.1 |
|  |  |  |  |  |  | ON380539.1 |
|  |  |  |  |  |  | ON380540.1 |
|  |  |  |  |  |  | ON400500.1 |
|  |  |  |  |  |  | ON963982.2 |
|  |  |  |  |  |  | OL310288.1 |
|  |  |  |  |  |  | MW521382.1 |
|  |  |  |  |  |  | MW656282.1 |
|  |  |  |  |  |  |  |
| 146749 | 148120* | 101381 | 102527* | ^NC_001659.2 | Unknown (NC_044944.1) | OR660089.1 |
|  |  |  |  |  | Unknown(NC_044945.1) | NC_044948.1 |
|  |  |  |  |  | Unknown(PP750552.1) | PP529961.1 |
|  |  |  |  |  | Unknown(MT956648.1) | PP355086.1 |
|  |  |  |  |  |  | OP479889.1 |
|  |  |  |  |  |  | OP718535.1 |
|  |  |  |  |  |  | OR449224.1 |
|  |  |  |  |  |  | OR290104.2 |
|  |  |  |  |  |  | OR227304.1 |
|  |  |  |  |  |  | OR180113.1 |
|  |  |  |  |  |  | OK358852.1 |
|  |  |  |  |  |  | OP672342.1 |
|  |  |  |  |  |  | ON409979.1 |
|  |  |  |  |  |  | ON409983.1 |
|  |  |  |  |  |  | OF448913.1 |
|  |  |  |  |  |  | OM105586.1 |
|  |  |  |  |  |  | ON380539.1 |
|  |  |  |  |  |  | ON380540.1 |
|  |  |  |  |  |  | ON400500.1 |
|  |  |  |  |  |  | ON963982.2 |
|  |  |  |  |  |  | OL310288.1 |
|  |  |  |  |  |  | MW521382.1 |
|  |  |  |  |  |  | MW656282.1 |
|  |  |  |  |  |  |  |
| 148166 | 148305 | 119771 | 119851 | ^MZ566623.1 | MN630494.2 | NC_044944.1 |
|  |  |  |  | NC_044946.1 |  | NC_044945.1 |
|  |  |  |  | OZ005801.1 |  | PP750552.1 |
|  |  |  |  | OZ003747.1 |  | MT956648.1 |
|  |  |  |  | ON409980.1 |  |  |
|  |  |  |  | MH025918.1 |  |  |
|  |  |  |  |  |  |  |
| 225333* | 225894* | 171169* | 171231* | ^MZ945536.1 | PP529961.1 | Unknown (OR387520.1) |
|  |  |  |  |  |  |  |
| 125005 | 125432 | 91526 | 91617 | ^OM249788.1 | LR881473.1 | Unknown (NC_044942.1) |
|  |  |  |  |  | NC_044943.1 | Unknown(NC_044941.1) |
|  |  |  |  |  |  | Unknown(MN270972.1) |
|  |  |  |  |  |  |  |
| 131778 | 131869 | 93010 | 93063 | MZ945537.1 | Unknown (PP828951.1) | OR371517.1 |
|  |  |  |  | MZ945536.1 | Unknown(NC_044941.1) | PP828951.1 |
|  |  |  |  |  | Unknown(NC_044942.1) |  |
|  |  |  |  |  | Unknown(NC_044955.1) |  |
|  |  |  |  |  | Unknown(NC_044956.1) |  |
|  |  |  |  |  | Unknown(NC_001659.2) |  |
|  |  |  |  |  | Unknown(OR371517.1) |  |
|  |  |  |  |  | Unknown(PP592890.1) |  |
|  |  |  |  |  | Unknown(OR420801.1) |  |
|  |  |  |  |  | Unknown(OR387520.1) |  |
|  |  |  |  |  | Unknown(MW736602.1) |  |
|  |  |  |  |  | Unknown(MN270972.1) |  |
|  |  |  |  |  | Unknown(MN270973.1) |  |
|  |  |  |  |  |  |  |
| 77998 | 78959 | 59418 | 60378 | ^NC_044953.1 | NC_044950.1 | NC_044941.1 |
|  |  |  |  |  |  | NC_044942.1 |
|  |  |  |  |  |  | NC_044943.1 |
|  |  |  |  |  |  | NC_044955.1 |
|  |  |  |  |  |  | NC_044956.1 |
|  |  |  |  |  |  | NC_001659.2 |
|  |  |  |  |  |  | PP810980.1 |
|  |  |  |  |  |  | OR371517.1 |
|  |  |  |  |  |  | PP348677.1 |
|  |  |  |  |  |  | OQ504954.1 |
|  |  |  |  |  |  | OQ504955.1 |
|  |  |  |  |  |  | OQ504956.1 |
|  |  |  |  |  |  | PP592890.1 |
|  |  |  |  |  |  | PP478517.1 |
|  |  |  |  |  |  | OR420801.1 |
|  |  |  |  |  |  | OR387520.1 |
|  |  |  |  |  |  | OM249788.1 |
|  |  |  |  |  |  | MZ945536.1 |
|  |  |  |  |  |  | MZ945537.1 |
|  |  |  |  |  |  | MW736602.1 |
|  |  |  |  |  |  | MZ202520.1 |
|  |  |  |  |  |  | LR881473.1 |
|  |  |  |  |  |  | MN270972.1 |
|  |  |  |  |  |  | MN270973.1 |
|  |  |  |  |  |  |  |
| 92808* | 92966* | 72601* | 72756* | NC_044952.1 | LR881473.1 | Unknown (PP529961.1) |
|  |  |  |  |  | NC_044941.1 | Unknown(NC_044948.1) |
|  |  |  |  |  | NC_044942.1 | Unknown(OR660089.1) |
|  |  |  |  |  | NC_044955.1 | Unknown(PP355086.1) |
|  |  |  |  |  | NC_044956.1 | Unknown(OP479889.1) |
|  |  |  |  |  | NC_001659.2 | Unknown(OP718535.1) |
|  |  |  |  |  | OR371517.1 | Unknown(OR449224.1) |
|  |  |  |  |  | PP592890.1 | Unknown(OR290104.2) |
|  |  |  |  |  | OR387520.1 | Unknown(OR227304.1) |
|  |  |  |  |  | MZ945536.1 | Unknown(OR180113.1) |
|  |  |  |  |  | MW736602.1 | Unknown(OK358852.1) |
|  |  |  |  |  | MN270972.1 | Unknown(OP672342.1) |
|  |  |  |  |  | MN270973.1 | Unknown(ON409979.1) |
|  |  |  |  |  |  | Unknown(OF448913.1) |
|  |  |  |  |  |  | Unknown(ON380539.1) |
|  |  |  |  |  |  | Unknown(ON380540.1) |
|  |  |  |  |  |  | Unknown(ON963982.2) |
|  |  |  |  |  |  | Unknown(OL310288.1) |
|  |  |  |  |  |  | Unknown(MW656282.1) |
|  |  |  |  |  |  |  |
| 21531* | 24480 | 15312* | 16108 | ^MN641877.2 | NC_044941.1 | Unknown (OR660089.1) |
|  |  |  |  |  | NC_044955.1 | Unknown(NC_044948.1) |
|  |  |  |  |  | NC_044956.1 | Unknown(PP529961.1) |
|  |  |  |  |  | OR371517.1 | Unknown(PP355086.1) |
|  |  |  |  |  | PP592890.1 | Unknown(OR290104.2) |
|  |  |  |  |  | OR387520.1 | Unknown(OR227304.1) |
|  |  |  |  |  | MW736602.1 | Unknown(OR180113.1) |
|  |  |  |  |  | MN270972.1 | Unknown(OK358852.1) |
|  |  |  |  |  | MN270973.1 | Unknown(ON409979.1) |
|  |  |  |  |  |  | Unknown(ON409983.1) |
|  |  |  |  |  |  | Unknown(OF448913.1) |
|  |  |  |  |  |  | Unknown(OM105586.1) |
|  |  |  |  |  |  | Unknown(ON380539.1) |
|  |  |  |  |  |  | Unknown(ON380540.1) |
|  |  |  |  |  |  | Unknown(ON963982.2) |
|  |  |  |  |  |  | Unknown(OL310288.1) |
|  |  |  |  |  |  | Unknown(MW521382.1) |
|  |  |  |  |  |  | Unknown(MW656282.1) |
|  |  |  |  |  |  |  |
| 28340 | 29001 | 16236 | 16896 | ^PP107957.1 | PP592890.1 | MN318203.3 |
|  |  |  |  | NC_044954.1 | NC_044941.1 |  |
|  |  |  |  |  | NC_044942.1 |  |
|  |  |  |  |  | NC_044955.1 |  |
|  |  |  |  |  | NC_044956.1 |  |
|  |  |  |  |  | PP810980.1 |  |
|  |  |  |  |  | OR371517.1 |  |
|  |  |  |  |  | PP348677.1 |  |
|  |  |  |  |  | OQ504954.1 |  |
|  |  |  |  |  | OQ504955.1 |  |
|  |  |  |  |  | OQ504956.1 |  |
|  |  |  |  |  | PP478517.1 |  |
|  |  |  |  |  | OR387520.1 |  |
|  |  |  |  |  | MZ945536.1 |  |
|  |  |  |  |  | MZ945537.1 |  |
|  |  |  |  |  | MW736602.1 |  |
|  |  |  |  |  | MN270972.1 |  |
|  |  |  |  |  | MN270973.1 |  |
|  |  |  |  |  |  |  |
| 131709 | 132246 | 107811 | 108227 | LR881473.1 | Unknown (OR420801.1) | OR371517.1 |
|  |  |  |  |  |  | NC_044956.1 |
|  |  |  |  |  |  | PP592890.1 |
|  |  |  |  |  |  |  |
| 48254 | 48709* | 33578 | 34018* | ^MN641876.2 | Unknown (MN318203.3) | NC_044946.1 |
|  |  |  |  |  |  | OZ005801.1 |
|  |  |  |  |  |  | OZ003747.1 |
|  |  |  |  |  |  | ON409980.1 |
|  |  |  |  |  |  | MZ566623.1 |
|  |  |  |  |  |  | MH025918.1 |
|  |  |  |  |  |  |  |
| 21546 | 24480 | 15686 | 16457 | ^MN336500.3 | MN270973.1 | Unknown (MW656282.1) |
|  |  |  |  | NC_044954.1 | NC_044948.1 | Unknown(NC_044941.1) |
|  |  |  |  | PP107957.1 | OR660089.1 | Unknown(NC_044955.1) |
|  |  |  |  | ON409981.1[T] | PP529961.1 | Unknown(NC_044956.1) |
|  |  |  |  |  | PP355086.1 | Unknown(OR371517.1) |
|  |  |  |  |  | OR290104.2 | Unknown(PP592890.1) |
|  |  |  |  |  | OR227304.1 | Unknown(OR387520.1) |
|  |  |  |  |  | OR180113.1 | Unknown(MW736602.1) |
|  |  |  |  |  | OK358852.1 | Unknown(MN270972.1) |
|  |  |  |  |  | ON409979.1 | Unknown(MN270973.1) |
|  |  |  |  |  | ON409983.1 |  |
|  |  |  |  |  | OF448913.1 |  |
|  |  |  |  |  | OM105586.1 |  |
|  |  |  |  |  | ON380539.1 |  |
|  |  |  |  |  | ON380540.1 |  |
|  |  |  |  |  | ON963982.2 |  |
|  |  |  |  |  | OL310288.1 |  |
|  |  |  |  |  | MW521382.1 |  |
|  |  |  |  |  | MW656282.1 |  |
|  |  |  |  |  |  |  |
| 48218* | 48713* | 35043* | 35538* | ^PP750552.1 | Unknown (NC_044945.1) | NC_044944.1 |
|  |  |  |  | MT956648.1 |  |  |
|  |  |  |  |  |  |  |
| 31596* | 33501 | 18138* | 19847 | ^NC_044949.1 | OR449224.1 | OR420801.1 |
|  |  |  |  |  | OL310288.1 |  |
|  |  |  |  |  |  |  |
| 206074 | 209870 | 168450 | 171446 | ^MN630494.2 | Unknown (MN336500.3) | MN394630.3 |
|  |  |  |  | MN641877.2 |  |  |
|  |  |  |  |  |  |  |
| 7593* | 7875* | 4275* | 4549* | ^NC_044954.1 | ON409980.1 | NC_044949.1 |
|  |  |  |  | PP107957.1 | NC_044946.1 |  |
|  |  |  |  |  | MH025918.1 |  |
|  |  |  |  |  |  |  |
| 91803 | 92164 | 74827 | 75062 | NC_044944.1 | OK358852.1 | MT956648.1 |
|  |  |  |  |  | NC_044951.1 |  |
|  |  |  |  |  |  |  |
| 28098* | 29001 | 17545* | 18434 | ^MN318203.3 | Unknown (ON409981.1) | OR371517.1 |
|  |  |  |  | MN641877.2[P] |  | NC_044941.1 |
|  |  |  |  |  |  | NC_044942.1 |
|  |  |  |  |  |  | NC_044955.1 |
|  |  |  |  |  |  | NC_044956.1 |
|  |  |  |  |  |  | PP592890.1 |
|  |  |  |  |  |  | OR387520.1 |
|  |  |  |  |  |  | MZ945536.1 |
|  |  |  |  |  |  | MZ945537.1 |
|  |  |  |  |  |  | MW736602.1 |
|  |  |  |  |  |  | MN270972.1 |
|  |  |  |  |  |  | MN270973.1 |
|  |  |  |  |  |  |  |
| 28149* | 29213 | 18512* | 19538 | ^MN394630.3 | Unknown (ON409981.1) | NC_044942.1 |
|  |  |  |  |  |  | NC_044941.1 |
|  |  |  |  |  |  | NC_044955.1 |
|  |  |  |  |  |  | NC_044956.1 |
|  |  |  |  |  |  |  |
| 174887 | 175914 | 142354 | 143174 | ^PP750552.1 | Unknown (NC_044950.1) | MN336500.3 |
|  |  |  |  | NC_044944.1 |  | MN394630.3 |
|  |  |  |  | NC_044945.1 |  |  |
|  |  |  |  | MT956648.1 |  |  |
|  |  |  |  |  |  |  |
| 26192 | 26549 | 14975 | 15096 | ^PP107957.1 | MW736602.1 | Unknown (ON409983.1) |
|  |  |  |  |  | NC_044941.1 | Unknown(OF448913.1) |
|  |  |  |  |  | NC_044955.1 | Unknown(OM105586.1) |
|  |  |  |  |  | NC_044956.1 | Unknown(ON380540.1) |
|  |  |  |  |  | OR371517.1 | Unknown(OL310288.1) |
|  |  |  |  |  | PP592890.1 |  |
|  |  |  |  |  | OR387520.1 |  |
|  |  |  |  |  | MN270972.1 |  |
|  |  |  |  |  | MN270973.1 |  |
|  |  |  |  |  |  |  |
| 5469* | 6802* | 3193* | 4270* | ^NC_044951.1 | Unknown (OP672342.1) | MZ945537.1 |
|  |  |  |  |  | Unknown(OR449224.1) | NC_044942.1 |
|  |  |  |  |  | Unknown(ON963982.2) | OR371517.1 |
|  |  |  |  |  |  | PP592890.1 |
|  |  |  |  |  |  | OR387520.1 |
|  |  |  |  |  |  | MN270972.1 |
|  |  |  |  |  |  | MN270973.1 |
