# Supplementary figures and images for: Host-similar fragments in the African swine fever virus genome: distribution, functions, and evolution
Source: Vet Res. 2025 May 27;56:108. doi: 10.1186/s13567-025-01539-3 (PMC12107907; doi:10.1186/s13567-025-01539-3)

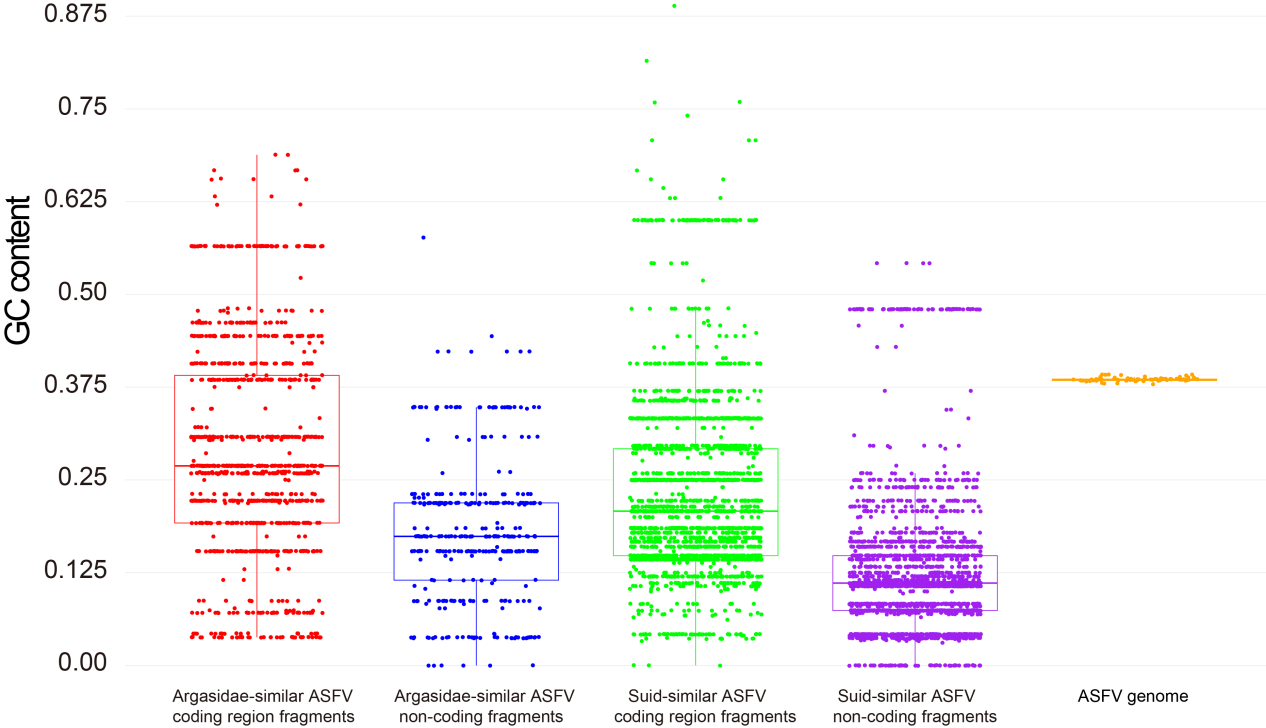

Supplement: Supplementary file 3 — Additional file 3: The GC content of host-similar fragments in the coding and non-coding regions of the ASFV genome. [file 13567_2025_1539_MOESM3_ESM.docx]

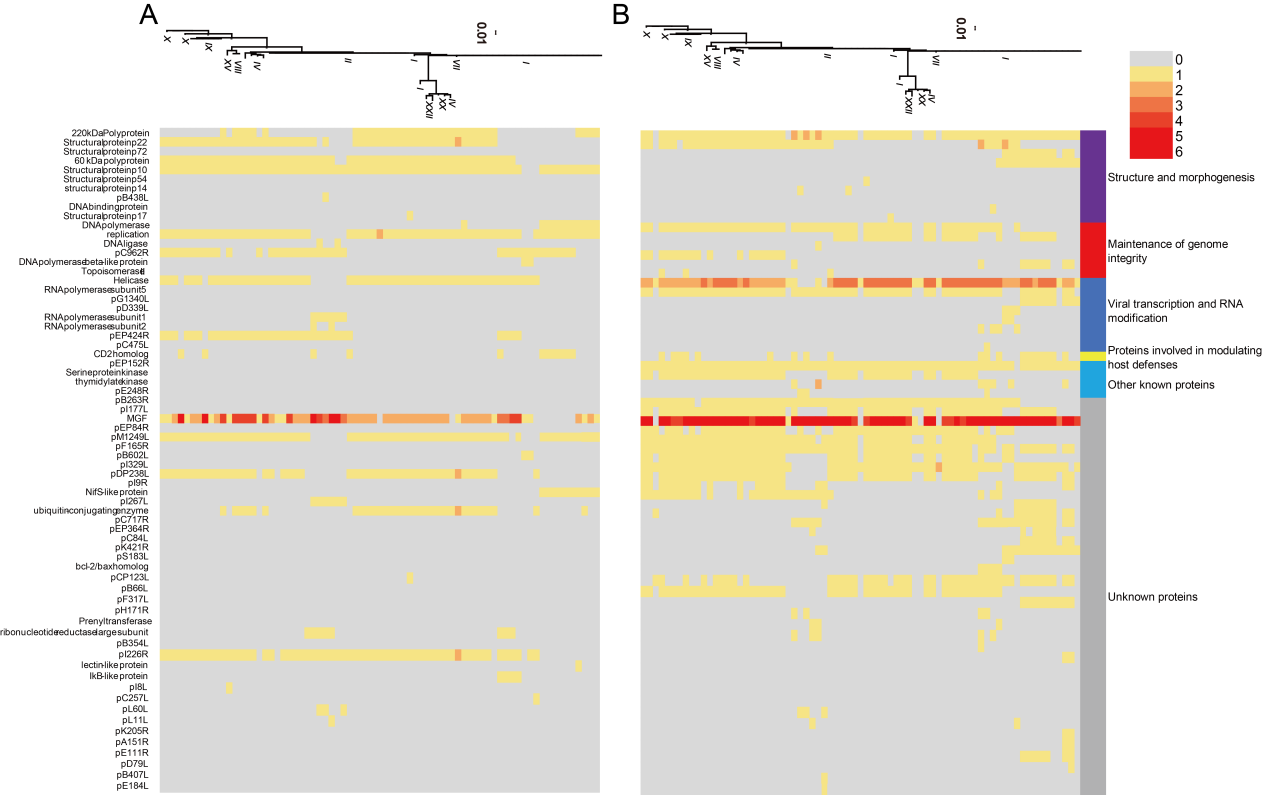

Supplement: Supplementary file 4 — Additional file 4: The functions of the ASFV proteins similar to those of the Argasidaeand suidproteins. [file 13567_2025_1539_MOESM4_ESM.docx]
